# P120-Catenin Isoforms 1 and 3 Regulate Proliferation and Cell Cycle of Lung Cancer Cells via β-Catenin and Kaiso Respectively

**DOI:** 10.1371/journal.pone.0030303

**Published:** 2012-01-20

**Authors:** Guiyang Jiang, Yan Wang, Shundong Dai, Yang Liu, Maggie Stoecker, Endi Wang, Enhua Wang

**Affiliations:** 1 Department of Pathology, First Affiliated Hospital and College of Basic Medical Sciences, China Medical University, Shenyang, China; 2 Department of Pathology, Duke University Medical Center, Durham, North Carolina, United States of America; Cincinnati Children's Hospital Medical Center, United States of America

## Abstract

**Background:**

The different mechanisms involved in p120-catenin (p120ctn) isoforms' 1/3 regulation of cell cycle progression are still not elucidated to date.

**Methods and Findings:**

We found that both cyclin D1 and cyclin E could be effectively restored by restitution of p120ctn-1A or p120ctn-3A in p120ctn depleted lung cancer cells. When the expression of cyclin D1 was blocked by co-transfection with siRNA-cyclin D1 in p120ctn depleted cells restoring p120ctn-1A or 3A, the expression of cyclin E was slightly decreased, not increased, implying that p120ctn isoforms 1 and 3 cannot up-regulate cyclin E directly but may do so through up-regulation of cyclin D1. Interestingly, overexpression of p120ctn-1A increased β-catenin and cyclin D1 expression, while co-transfection with siRNA targeting β-catenin abolishes the effect of p120ctn-1A on up-regulation of cyclin D1, suggesting a role of β-catenin in mediating p120ctn-1A's regulatory function on cyclin D1 expression. On the other hand, overexpression of p120ctn isoform 3A reduced nuclear Kaiso localization, thus decreasing the binding of Kaiso to KBS on the cyclin D1 promoter and thereby enhancing the expression of cyclin D1 gene by relieving the repressor effect of Kaiso. Because overexpressing NLS-p120ctn-3A (p120ctn-3A nuclear target localization plasmids) or inhibiting nuclear export of p120ctn-3 by Leptomycin B (LMB) caused translocation of Kaiso to the nucleus, it is plausible that the nuclear export of Kaiso is p120ctn-3-dependent.

**Conclusions:**

Our results suggest that p120ctn isoforms 1 and 3 up-regulate cyclin D1, and thereby cyclin E, resulting in the promotion of cell proliferation and cell cycle progression in lung cancer cells probably via different protein mediators, namely, β-catenin for isoform 1 and Kaiso, a negative transcriptional factor of cyclin D1, for isoform 3.

## Introduction

In cancer cells, β-catenin, a key factor of the canonical Wnt signaling pathway, accumulates in the cytoplasm and then translocates into the nucleus to form a complex with the transcription factor Lef/TCF (Lef, lymphoid enhancer factor; TCF, T-cell factor protein), which subsequently activates Wnt-responsive genes, including cyclin D1 [1]. Previously, we have reported that one of the p120ctn isoforms, p120ctn-1, could up-regulate β-catenin expression, whereas another p120ctn isoform, p120ctn-3, could not [2]–[3]. Interestingly, both p120ctn isoforms 1 and 3 could affect cell proliferation and cell cycle progression, which are known to be mediated by cyclin D1 [2]–[4]. It is thus reasonable to speculate that p120ctn-1 regulates cell proliferation and cell cycle through β-catenin induced up-regulation of cyclin D1. However, the underlying molecular mechanism by which p120ctn-3 regulates cell proliferation and cell cycle progression is still unclear at the present time.

Our previous study demonstrated that Kaiso, a nuclear BTB/POZ-ZF (BTB, Broad complex, Tramtrack, Bric à brac; POZ, poxvirus and zinc finger; ZF, zinc finger) transcription factor, could bind to p120ctn in lung cancer tissue and lung cancer cells [5]. Although known to be a component of the Kaiso/p120ctn complex, each individual p120ctn isoform might possess a different affinity while binding to Kaiso [6]. Of interest, the promoter region of cyclin D1 contains KBS (Kaiso-binding sites), a consensus DNA sequence that could be recognized by Kaiso [7]–[8]. Therefore, cyclin D1 may also be a potential downstream target gene of Kaiso [9]–[10].

Since the binding domain of Kaiso with p120ctn is completely overlapped with the specific DNA sequence of KBS element on the cyclin D1 promoter [9]–[11], we hypothesize that p120ctn-3 could compete with KBS on cyclin D1 gene to bind to Kaiso and abrogate Kaiso-mediated repression of cyclin D1, thus enhancing the expression of cyclin D1 as well as cyclin E, which would ultimately promote cell cycle progression. To test our hypothesis in the present study, we reconstituted p120ctn-1A and 3A respectively in p120ctn depleted cells and overexpressed or knocked down Kaiso or β-catenin to test the effects on expression of cyclin D1 and cyclin E. The objective was to investigate the potential different molecular mechanisms by which p120ctn-1 and 3 regulate cell proliferation and the cell cycle in lung cancer cells. Two deletion mutants of p120ctn isoform 1, which vary in their N-terminal structures, were constructed to study the different functions of p120ctn-1 and 3.

## Results

### Both p120ctn 1A and 3A could up-regulate cyclin D1 and cyclin E expression, affecting cell proliferation and cell cycle progression in lung cancer cells

**Figure 1:**
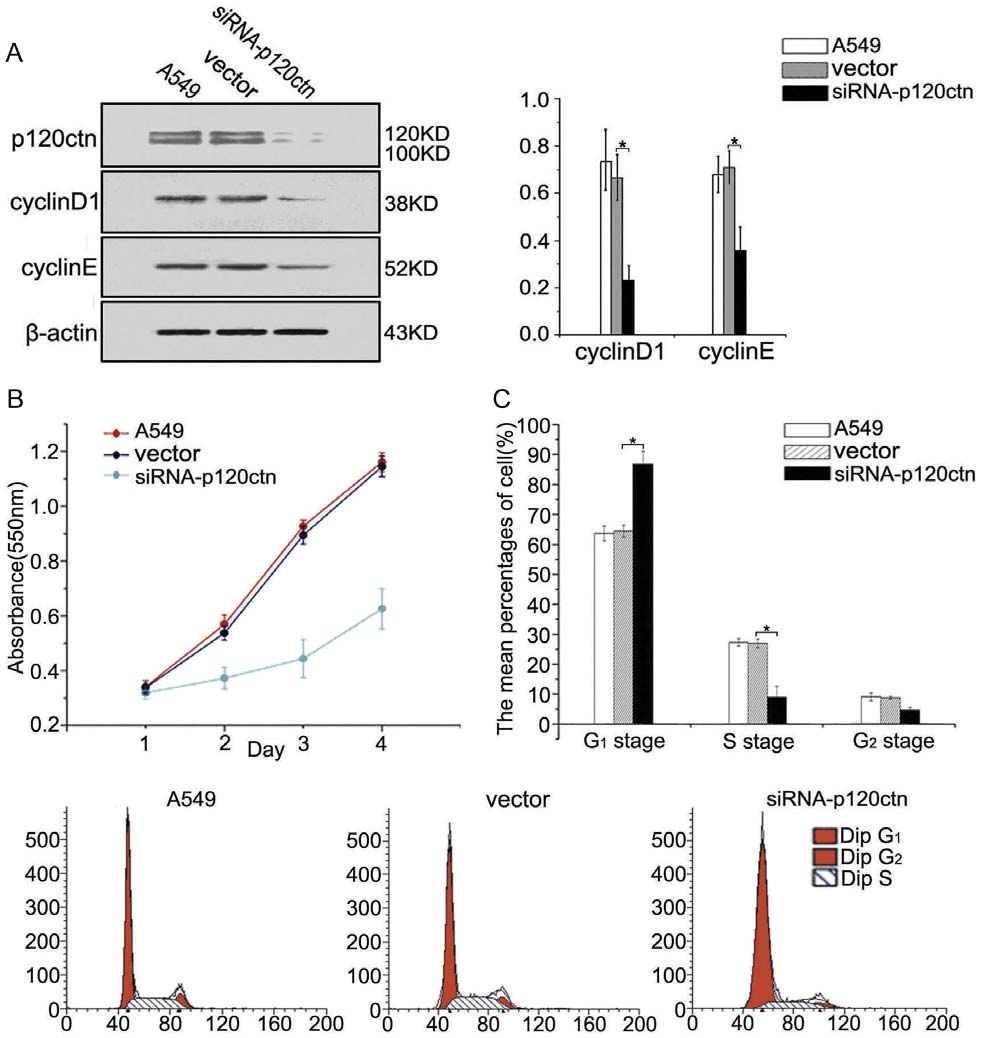
Cyclin D1 and cyclin E expression were significantly decreased in A549 cells with knocked down p120ctn. (**A**) The results of western blot analysis showed that proteins cyclin D1 (*, *p* = 0.001) and cyclin E (*, *p* = 0.004) were significantly decreased after p120ctn was knocked down in A549 cells. (**B** and **C**) The results of MTT and flow cytometry showed suppressed cell proliferative ability, increased G_1_ phase cells (*, *p* = 0.001) and reduced S phase cells (*, *p* = 0.001) were detected in A549 cells with knocked down p120ctn. All comparisons were made to the groups of A549 cells or the cells transfected with vector alone.

**Figure 2:**
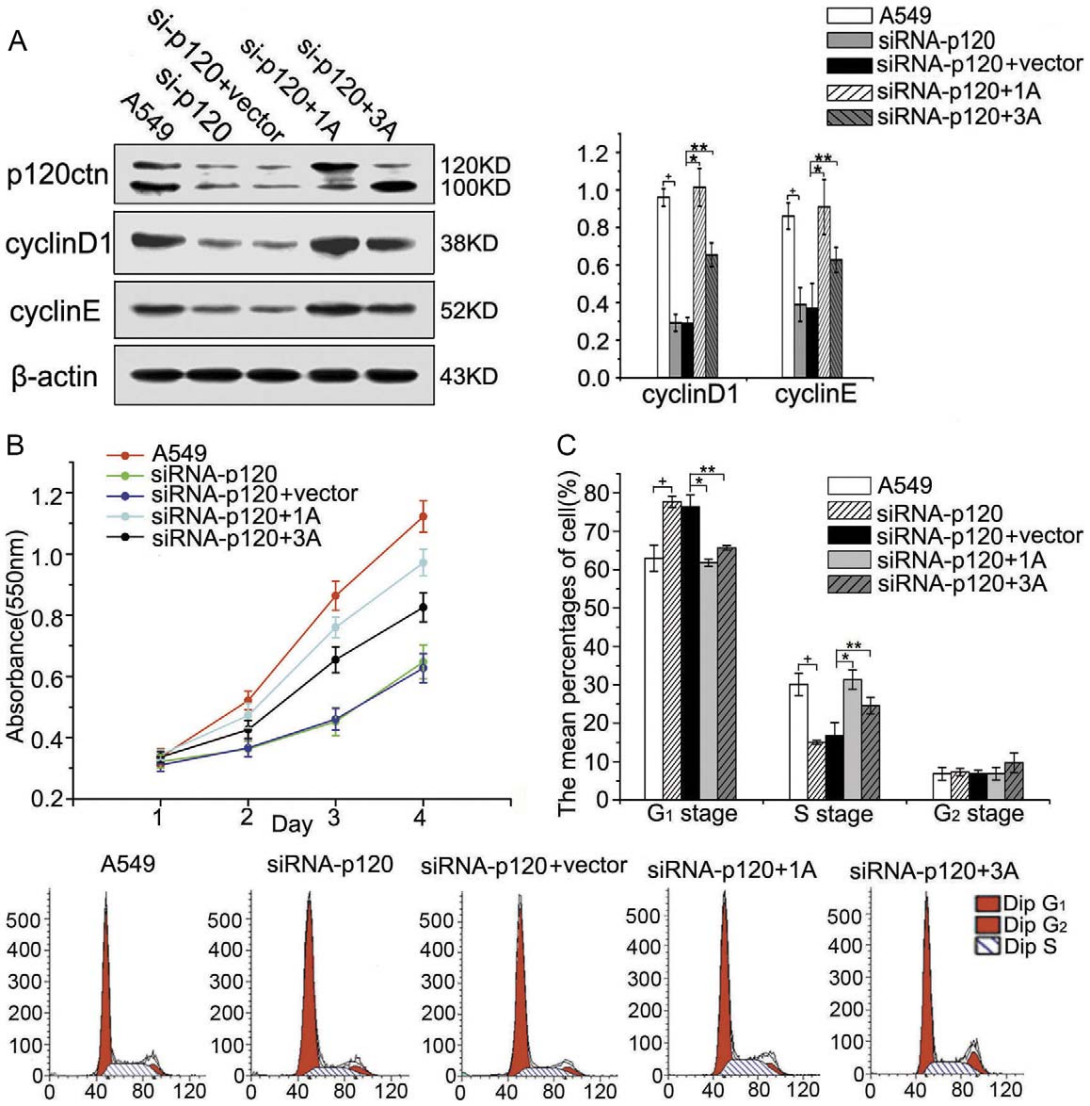
Restitution of p120ctn-1A or p120ctn-3A in p120ctn depleted A549 cells could restore cyclin D1 and cyclin E. (**A**) p120ctn-1A or 3A plasmids were transfected in A549 cells depleted of p120ctn (si-p120+1A or si-p120+3A), showing the significantly recovered protein levels of cyclin D1 (*, *p*<0.001, **, *p*<0.001) and cyclin E (*, *p*<0.001, **, *p*<0.01). (**B** and **C**) The results of MTT and flow cytometry showed that cell proliferation was effectively restored (*p*<0.01), G_1_ phase cells were significantly decreased (*, *p* = 0.001, **, *p* = 0.004) and S phase cells were significantly increased (*, *p* = 0.004, **, *p* = 0.028) after p120ctn depleted A549 cells were transfected with p120ctn-1A or 3A. All the comparisons are made to the groups of A549 cells and the cells transfected with vector alone.

Whether p120ctn acts as a tumor suppressor or a metastasis promoter largely depends on if the expression of E-cadherin is down-regulated, completely lost from the cell-cell junction or intact [12]. Therefore, it is crucial to determine if E-cadherin is localized on the membrane of human lung cancer cells. In this study, we confirmed that no visible membranous localization of either p120ctn-1/3 or E-cadherin proteins in A549 or SPC cell lines (Figure S1). They instead showed predominantly cytoplasmic distribution. After p120ctn was knocked down in A549 cells, the expressions of cyclin D1 and cyclin E were significantly reduced at levels of both protein (Figure 1A) and transcript (Figure S2A). In accordance, cell proliferation was effectively suppressed (*p*<0.01, Figure 1B), indicated by more G_1_ phase cells and less S phase cells detected (*p* = 0.001, Figure 1C). Furthermore, cyclin D1 and cyclin E could be effectively restored by restitution of p120ctn-1A or p120ctn-3A in p120ctn depleted A549 cells (Figure 2A and Figure S2B), and the cell proliferation and cell cycle could also be restored, presumably corresponding to the repletion of cyclin D1 and cyclin E (*p*<0.05, Figure 2B and C). Similar results were obtained in SPC-K2 cells in which p120ctn expression was constitutionally depleted (Figure S3).

**Figure 3:**
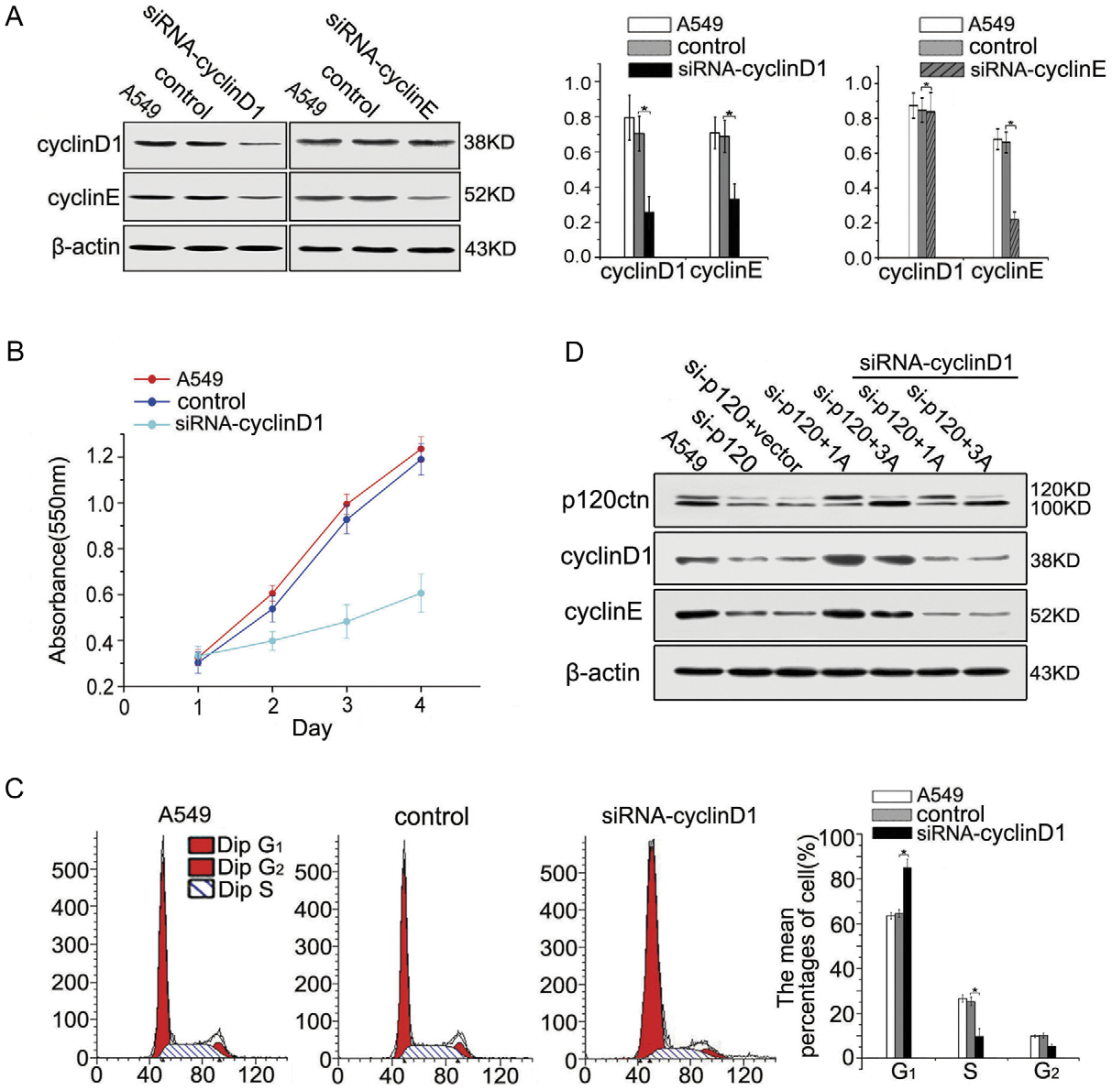
p120ctn promotes cell cycle progression by up-regulating cyclin D1, which subsequently enhances cyclin E expression. (**A**) Cyclin D1 depletion by siRNA in A549 cells led to reduced cyclin E expression, but conversely, the expression of cyclin D1 was not significantly changed (*p* = 0.944) in cells with knocked down cyclin E by siRNA, suggesting a unidirectional regulatory relationship between cyclin D1 and cyclin E. (**B** and **C**) Cyclin D1 depletion suppressed cell proliferation (*p*<0.01), elevated G_1_ phase cells (*p* = 0.016) and decreased S phase cells (*p* = 0.009).(**D**) After co-transfection of siRNA-cyclin D1 with p120ctn isoform 1 or 3 in the cells depleted of p120ctn for 48 hours, the expression of cyclin E was not increased but was slightly decreased. All these results suggest that p120ctn promotes cell cycle progression by up-regulating cyclin D1, which subsequently enhances expression of cyclin E. The comparison is made to the control group of cells transfected with non-targeted siRNA or vector alone.

We then found that cyclin D1 depletion by siRNA in A549 cells also led to the reduction of cyclin E expression (Figure 3A and Figure S4A), suppression of cell proliferation (*p*<0.01, Figure 3B), elevation of G_1_ phase cells and decrease in S phase cells (*p* = 0.009, Figure 3C), which were comparable to the interference of p120ctn. In contrast, no change in cyclin D1 expression was observed when cyclin E was depleted by siRNA (Figure 3A and Figure S4A), suggesting a unidirectional regulatory relationship between cyclin D1 and cyclin E. Interestingly, when siRNA-cyclin D1 was co-transfected with p120ctn isoform 1 or 3 into the cells depleted of p120ctn, the expression of cyclin E was not increased but slightly decreased (Figure 3D and Figure S4B), and a similar phenomenon was observed when cyclin D1 was blocked by monoclonal antibody incubation (100 ng/ml, 48 h) in p120ctn reconstituted cells (Figure S5). This finding implies that p120ctn isoforms 1 and 3 could not up-regulate cyclin E directly, but might do so through up-regulation of cyclin D1. All these results suggest that p120ctn promotes cell cycle progression by up-regulating cyclin D1, which subsequently enhances the expression of cyclin E.

### p120ctn isoform 1 could up-regulate cyclin D1 expression through β-catenin

**Figure 4:**
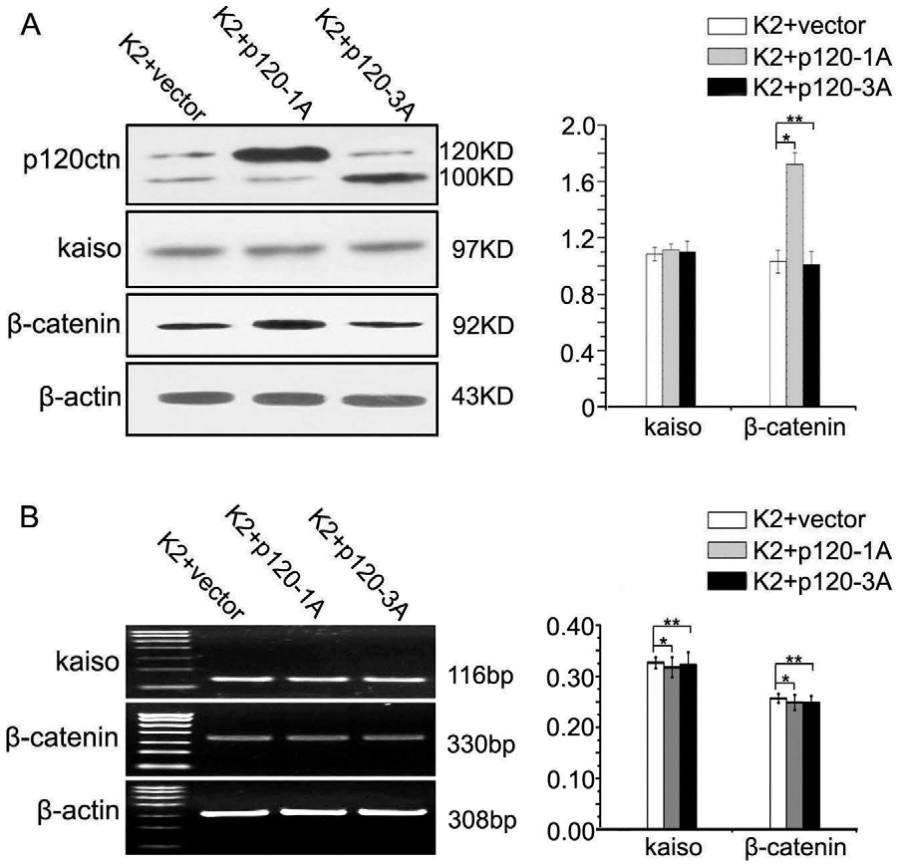
p120ctn-1A, but not p120ctn-3A, increases the protein expression of β-catenin. SPC-K2 cells were transfected with p120ctn-1A or 3A respectively, and the expression of β-catenin and kaiso was measured by western blot (**A**) and RT-PCR (**B**). The protein expression of β-catenin was significantly increased (*, *p* = 0.001) in the cells transfected with p120ctn-1A cDNA, but it showed no significant change (**, *p* = 0.769) in p120ctn-3A overexpressing SPC-K2. Neither p120ctn-1 nor 3 could affect the transcription of *β-catenin* (*p = 0.463, **p = 0.401) or the expression of Kaiso. All the comparisons are made to the group of cells transfected with vector alone.

Since we have confirmed that both p120ctn-1A and p120ctn-3A could up-regulate cyclin D1, the question was raised whether the underlying molecular mechanisms were the same between the two isoforms. To answer this question, p120ctn-1A and p120ctn-3A were respectively transfected into p120ctn knocked down SPC cells, which were designated as SPC-K2. Overexpression of p120ctn-1A could enhance β-catenin expression (*p = 0.001, Figure 4A), but overexpression of p120ctn-3A had essentially no effect on β-catenin protein (**p = 0.769, Figure 4A). Of interest, neither p120ctn-1 nor 3 appeared to have effect on the transcription of β-catenin (*p = 0.463, **p = 0.401, Figure 4B).

**Figure 5:**
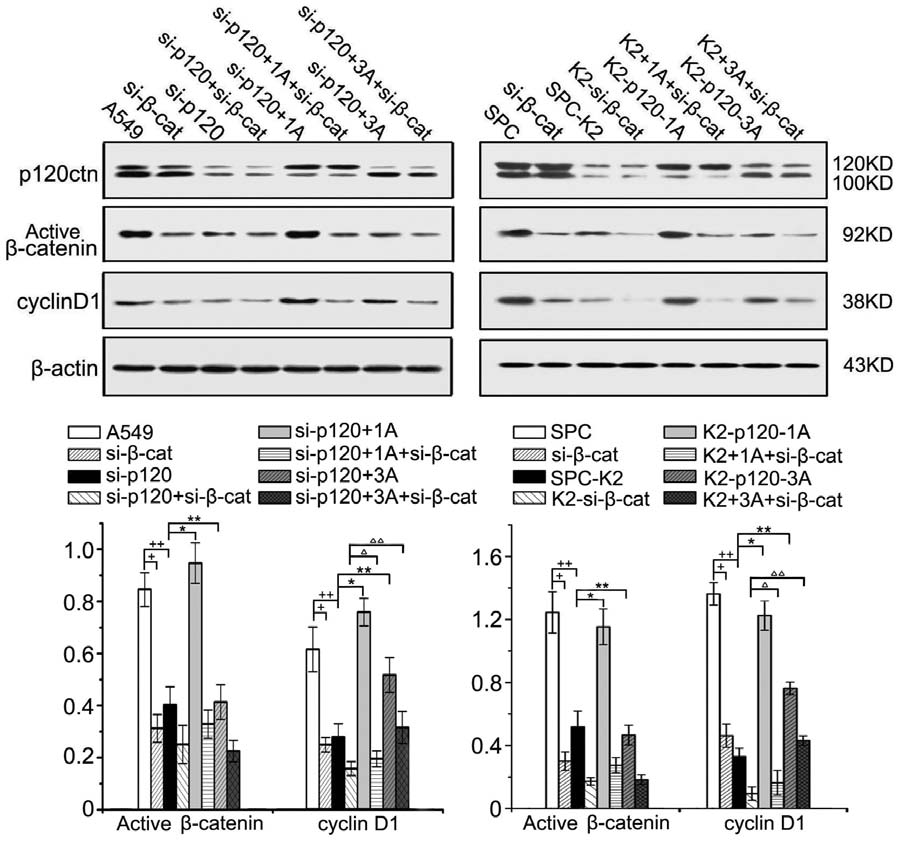
p120ctn isoform 1 could up-regulate cyclin D1 expression through β-catenin. The expression of active β-catenin and cyclin D1 were significantly reduced in A549 cells (β-catenin, + *p*<0.001, ++ *p*<0.001, cyclin D1, + *p* = 0.003, ++ *p* = 0.004) when β-catenin or p120ctn was interfered (si-β-cat or si-p120). The expression of active β-catenin (* *p* = 0.001) and cyclin D1 (* *p*<0.001) rebounded when p120ctn-1A expression was reconstituted by p120ctn-1A cDNA transfection (si-p120+1A). Co-transfection of p120ctn-1A and the siRNA targeting β-catenin (si-p120+1A+si-β-cat) showed no change of cyclin D1 (^Δ^ *p* = 0.248) expression, suggesting that p120ctn-1A up-regulates cyclin D1 via β-catenin. Restoration of p120ctn-3A (si-p120+3A) did not increase the expression of active β-catenin (** *p* = 0.891) but could still restore the expression of cyclin D1 (** *p* = 0.001), implying that p120ctn-3 up-regulates expression of cyclin D1 independent of β-catenin. Co-transfection of p120ctn-3A and the siRNAs targeting p120ctn/β-catenin (si-p120+3A+si-β-cat) could significantly increase cyclin D1 expression in A549 cell line in comparison with the group of p120ctn/β-catenin siRNA transfection alone (si-p120+si-β-cat, ^ΔΔ^ *p*<0.01), whereas, co-transfection of p120ctn-3A seems to have no impact on the levels of active β-catenin. Similar results were obtained in SPC cells. Note, although cyclin D1 expression is restored by restitution of either p120ctn 1A or p120ctn 3A, the rescue effect is more prominent by p120ctn 1A than by p120ctn 3A. The difference between the group si-p120+3A and the group si-p120+3A+si-β-cat may be explained by an effect of residual endogenous β-catenin in the former. This effect of residual endogenous β-catenin can also be seen in the difference between si-p120 and si-p120+si-β-cat in both cell lines. A synergistic effect resulting in additional decrease in both β-catenin and cyclin D1 can be seen in the latter group.

As shown in Figure 5, down-regulation of β-catenin by siRNA (si-β-cat) resulted in reduced cyclin D1 expression in A549, and knocking-down p120ctn (si-p120) resulted in reduced active β-catenin and cyclin D1. Moreover, active β-catenin and cyclin D1 levels could be restored by transfecting p120ctn-1A (si-p120+1A). However, cyclin D1 expression was not restored by co-transfecting p120ctn-1A and siRNA -β-catenin (si-p120+1A+si-β-cat), suggesting that p120ctn-1A up-regulated cyclin D1 via β-catenin. Transfection of p120ctn-3A in the cells depleted of p120ctn (si-p120+3A) could not up-regulate active β-catenin levels but could still significantly restore cyclin D1 expression, implying that p120ctn-3 up-regulates expression of cyclin D1 independent of β-catenin. In addition, when p120ctn-3A was co-transfected into A549 cells depleted of both p120ctn and β-catenin (si-p120+3A+si-β-cat), cyclin D1 expression was significantly increased in comparison with the group depleted of p120ctn and β-catenin without restituting p120ctn-3A (si-p120+si-β-cat); whereas, no change in active β-catenin level was observed in p120ctn and β-catenin dual depleted cells, despite their apparent restitution of p120ctn-3A (si-p120+3A+si-β-cat). Similar results were obtained in experiments carried out in SPC cells (Figure 5). Two interesting findings were noted in our study. 1). The effect of p120ctn-1A on cyclin D1 expression seemed to be more prominent than that of p120ctn-3A, since the expression of cyclin D1 induced by restitution of p120ctn-3A was always lower than that induced by restitution of p120ctn-1A or even lower than untreated cells; 2). The restoration of cyclin D1 induced by restitution of p120ctn-3A in the cells depleted of both p120ctn and β-catenin (si-p120ctn+3A+si-β-cat) seemed to be incomplete when compared with the cells depleted of p120ctn alone (si-p120+3A). The difference between these two groups might be explained by the endogenous β-catenin in the latter, which was suppressed synergistically by siRNA-β-catenin in the former.

β-catenin distribution in A549 and SPC cells was observed in both nucleus and cytoplasm under confocal laser microscopy. Its signal became significantly weaker in corresponding subcellular zones when p120ctn was depleted. Overexpression of p120ctn-1A resulted in a stronger β-catenin signal. In contrast, p120ctn-3A has no appreciable effect on expression of β-catenin (Figure S6).

**Figure 6:**
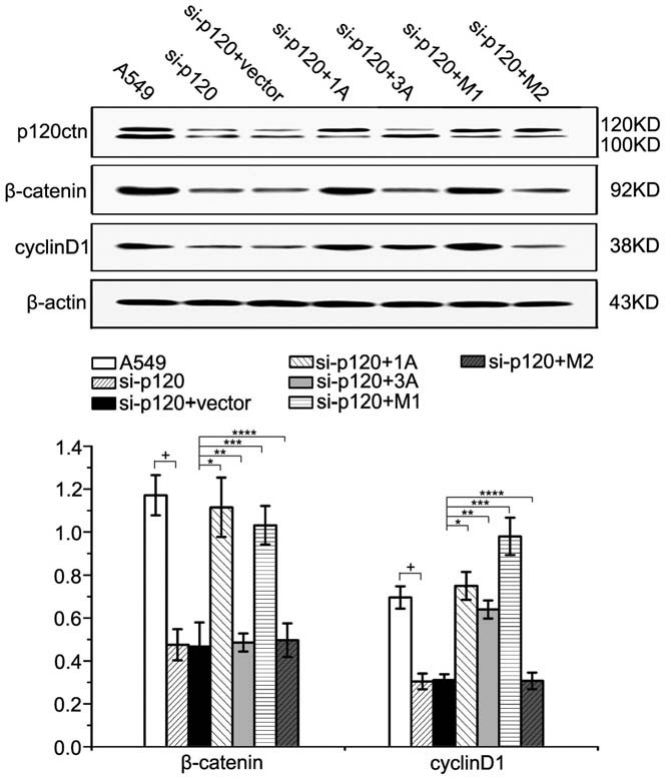
N-56-101 amino acid residues of p120ctn-1 are essential for up-regulating β-catenin. Two deletion mutants of p120ctn-1, M1 and M2 were transfected in p120ctn depleted A549 cells. The results showed that M1 could up-regulate β-catenin (*** *p*<0.001), while M2 could not (**** *p* = 0.175). M1 could up-regulate the expression of cyclin D1 (*** *p*<0.001) but M2 could not (**** *p* = 0.906). Therefore, it may be due to deletion of N-56-101 amino acid residues for p120ctn-3 to fail to regulate β-catenin. All the comparisons are made to the group of cells co-transfected with vector alone.

To further test the function of these two isoforms, we transfected two deletion mutants of p120ctn-1, M1 and M2, in the p120ctn depleted A549 cell (Figure 6). The results showed that M1 could up-regulate β-catenin but M2 could not, implying that N-56-101 amino acid residues of p120ctn-1 are essential for up-regulating β-catenin. Therefore, it may be due to deletion of N-56-101 amino acid residues for p120ctn-3 to fail to regulate the level of β-catenin protein.

### p120ctn isoform 3 could up-regulate cyclin D1 expression by regulating the nuclear/cytoplasmic shuttle of Kaiso

**Figure 7:**
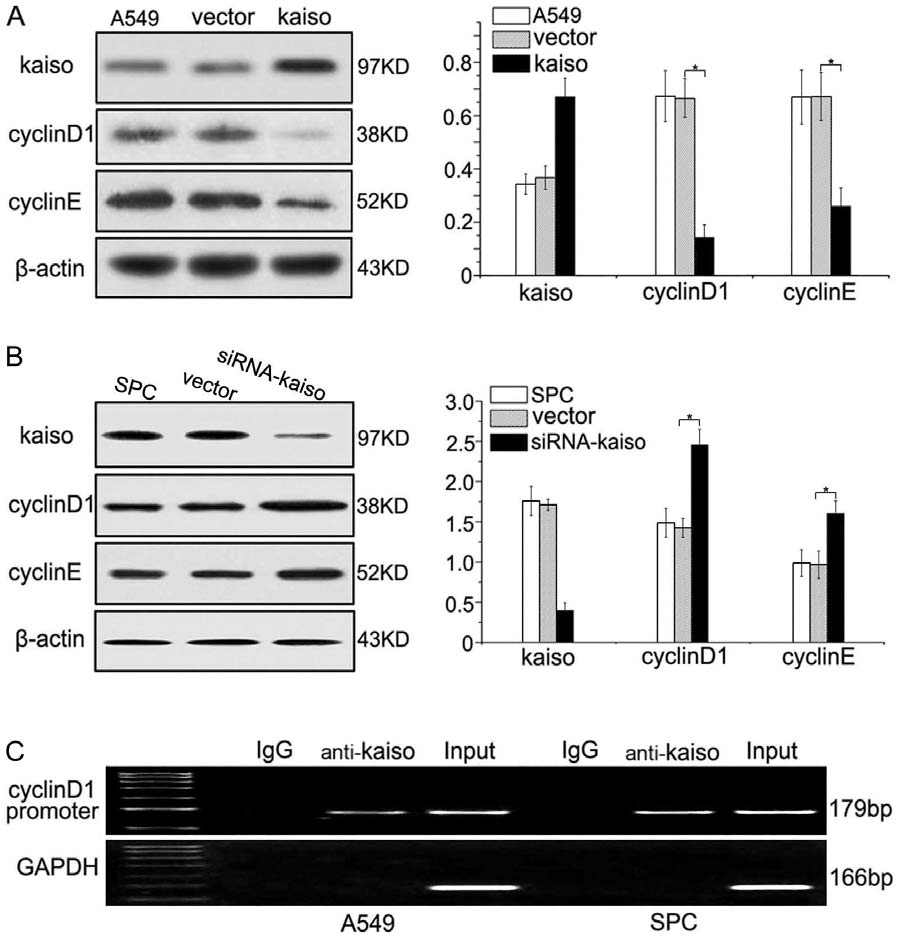
Cyclin D1 is one of downstream target genes of Kaiso. (**A**) Western blot analyses show the increased expression of Kaiso in A549 cells transfected with Kaiso cDNA. Kaiso overexpression remarkably down-regulated the expression of cyclin D1 (p = 0.000) and cyclin E (p = 0.003). (**B**) Western blot analyses show reduced expression of Kaiso in SPC cells transfected with Kaiso siRNA. Kaiso interference significantly increased the expression of cyclin D1 (p = 0.001) and cyclin E (p = 0.012). Overall findings suggest that Kaiso could regulate the expression of cyclin D1 and cyclin E. (**C**) Chromatin immunoprecipitation (ChIP) assay confirmed the Kaiso monoclonal antibody could precipitate the cyclin D1 gene promoter fragment containing KBS. Kaiso could bind to KBS of cyclin D1 promoter. All the comparisons are made to the group of cells transfected with vector alone.

To test if Kaiso has a role in regulating expression of cyclin D1, we transiently transfected Kaiso into A549 cells, which express only a minor amount of Kaiso. Kaiso overexpression led to reduced cyclin D1 and cyclin E expression (Figure 7A and Figure S7A). Correspondingly, when Kaiso was knocked down by siRNA, there was enhanced cyclin D1 and cyclin E expression in SPC cells, which express relatively high levels of Kaiso (Figure 7B and Figure S7B).

Using BLAST to find the cyclin D1 promoter sequence (GenBank: AY439218.1), we identified the specific Kaiso binding sequence (KBS, TCCTGCNA), which lies in the region of −1059 bp to −1066 bp. The ChIP assay was then performed and showed that Kaiso was associated with KBS on the cyclin D1 promoter in both A549 and SPC cells (Figure 7C).

**Figure 8:**
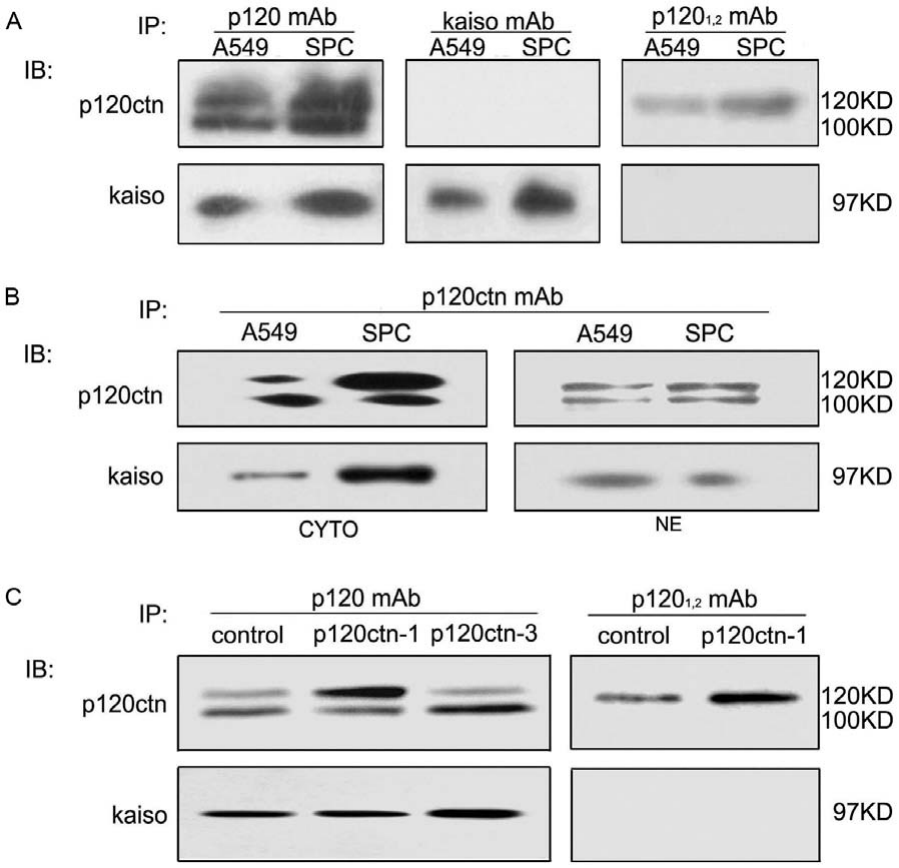
Only p120ctn-3 could bind to Kaiso in vivo. (**A** and **B**) Co-immunoprecipitation assays showed that p120ctn monoclonal antibody (the lower panel of left column in Figure 8A), but not specific p120ctn-1,2 monoclonal antibody (6H11) (the lower panel of right column in Figure 8A), can effectively precipitate Kaiso protein both in the nucleus and cytoplasm (Figure 8B). However, Kaiso monoclonal antibody cannot effectively precipitate p120ctn (the upper panel of middle column in figure 8A). (**C**) Co-immunoprecipitation with sufficient p120ctn mAb after overexpression of p120ctn-1 or 3 isoform showed that overexpression of p120ctn-3 led to more Kaiso protein precipitated in A549 cells. No change was detected when p120ctn isoform 1 was overexpressed, compared with the control. Specific p120ctn-1, 2 monoclonal antibodies (6H11) could not precipitate Kaiso when p120ctn isoform 1 was significantly increased. Note the presence of p120ctn after coprecipitation with 6H11 but absence of kaiso in the precipitate, suggesting there is no significant interaction between p120 isoform 1, 2 and kaiso. Since there are mainly p120ctn isoforms 1 and 3 in both of the lung cancer cell lines, we thought that Kaiso might bind to p120ctn isoform 3 but not to p120ctn isoform 1 in vivo.

Co-immunoprecipitation was carried out following cell fractionation. The assays revealed that the p120ctn monoclonal antibody could effectively precipitate Kaiso protein in both cytoplasm and nucleus, while the specific p120ctn-1, 2 monoclonal antibodies (6H11) could not do so (Figure 8A and B). When we repeated the co-immunoprecipitation after overexpression of p120ctn isoforms 1 and 3, respectively, it was shown that overexpression of p120ctn-3A led to more Kaiso protein precipitated by the p120ctn monoclonal antibody, but there was no change after overexpression of p120ctn-1A compared with the control. When the specific p120ctn-1, 2 monoclonal antibodies (6H11) were used, Kaiso could still not be precipitated after p120ctn-1A was significantly increased (Figure 8C). Since there are mainly p120ctn isoform 1 and 3 in both of the lung cancer cell lines, we thought that Kaiso might bind to p120ctn isoform 3, but not to p120ctn isoform 1 in vivo given the aforementioned results.

Kaiso is mainly localized in the cytoplasm of SPC cells, which express relatively high levels of p120ctn. In contrast, almost all Kaiso proteins are present with nuclear distribution in SPC-K2 cells, which have p120ctn depleted. Different effects of two p120ctn isoforms on Kaiso subcellular localization were examined following restitution of two isoforms respectively in SPC-K2 cells. Restoration of p120ctn isoform 3 by transfection of p120ctn-3A induced Kaiso's cytoplasmic distribution, whereas Kaiso remained essentially in nuclei of SPC-K2 cells when p120ctn isoform 1 was restored by transfection of p120ctn-1A (Figure S8A). Similarly, Kaiso was predominantly localized in the nuclei of A549 cells, which express only a minor amount of p120ctn. Overexpression of p120ctn-1A did not significantly alter the distribution of Kaiso, while overexpression of p120ctn-3A resulted in Kaiso export from the nucleus (Figure S8B). These findings suggest that a high level of p120ctn-3A may be able to promote the nuclear export of Kaiso, but p120ctn-1A does not appear to have this function.

Furthermore, when A549 cells transfected with p120ctn-3A were incubated with LMB to block the nuclear export of p120ctn isoform 3, Kaiso was noted to be confined in the nucleus along with p120ctn isoform 3. Correspondingly, overexpression of p120ctn-3A by transfecting NLS-p120ctn-3A, a p120ctn-3A nuclear target localization plasmid, into SPC-K2 cells resulted in a distribution of Kaiso predominantly in the nucleus (Figure S8B).

**Figure 9:**
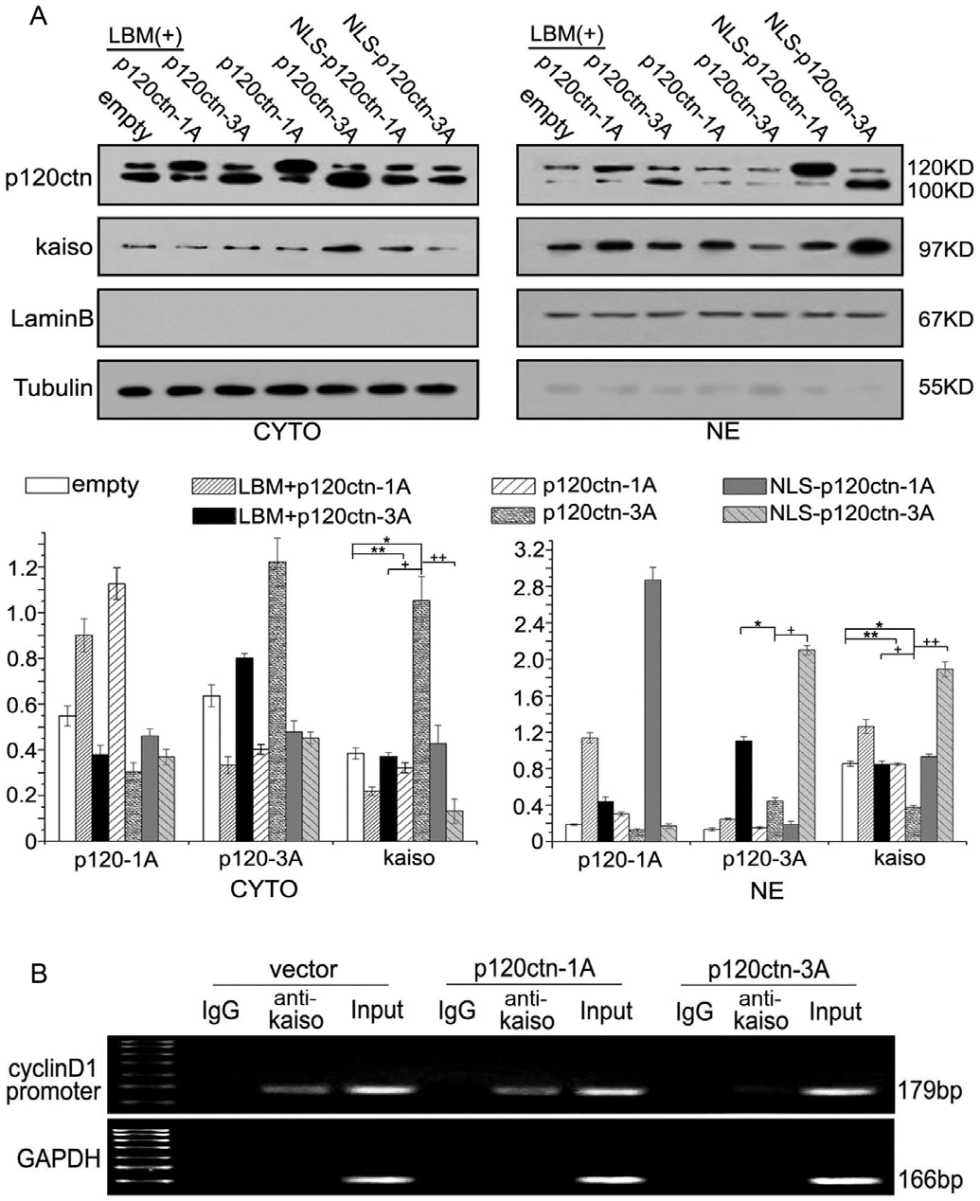
p120ctn-3 inhibit Kaiso from binding to cyclin D1 promoter by regulating the nuclear/cytoplasmic shuttle of Kaiso. (**A**) Transfection of p120ctn-3A in A549 cells significantly increased Kaiso in the cytoplasm (CYTO, *, p = 0.000) and reduced Kaiso in the nucleus (NE, *, p = 0.000). However, no significant difference of subcellular localization of Kaiso was found between p120ctn-1A overexpression cells and control cells (CYTO, **, p = 0.135, NE, **, p = 0.774). These results suggest that a high level of p120ctn-3A may be able to promote the nuclear export of Kaiso, but p120ctn-1A does not appear to have this function. Incubating cells transfected with p120ctn-3A with LMB or transfecting NLS-p120ctn-3A in A549 cells increased nuclear p120ctn-3A (*, p = 0.000, or +, p = 0.000) and nuclear Kaiso (NE, +, p = 0.000 or ++, p<0.001), but reduced cytoplasmic Kaiso (CYTO, +, p = 0.000 or ++, p = 0.002) compared to the cells with only overexpressed p120ctn-3A, implying that the nuclear export of Kaiso is likely to be p120ctn-3-dependent. (**B**) p120ctn-1A overexpression did not change the binding of Kaiso to KBS on the cyclin D1 promoter, whereas p120ctn-3A overexpression significantly reduced the binding of Kaiso to KBS on the cyclin D1 promoter.

We further confirmed the cytoplasmic and nuclear distribution of p120ctn and Kaiso by Western blotting after cell fractionation (Figure 9A). The results were consistent with those observed in the immunofluorescence experiments described above.

A few interesting results were observed in subsequent ChIP assays. Overexpression of p120ctn-3A significantly reduced the binding of Kaiso to KBS, while overexpression of p120ctn-1A did not change the binding (Figure 9B).

In summary, the above findings suggest that p120ctn-3A likely up-regulate cyclin D1 by facilitating nuclear export of kaiso, and thus, abolishing the negative effect of Kaiso on cyclin D1 gene expression.

## Discussion

We demonstrated that depletion of p120ctn in A549 and SPC cells, which show no membranous p120ctn localization, resulted in increased cells in G1 phase and decreased cells in S phase as well as reduced cell proliferation, suggesting p120ctn can affect cell cycle progression and cell proliferation. Since reduced expression of cyclin D1 and cyclin E was observed in the p120ctn depleted cells, it was thought that p120ctn might regulate cell cycle progression and cell proliferation by altering expression of cyclin D1 and cyclin E, the essential modulators of the G0/G1-S checkpoint [13]. Nicolas T. et al. reported that p120ctn isoform 3 could affect cell cycle and cell proliferation by stabilizing cyclin E [4]. In contrast, our data suggest a different mechanism by the fact that increased expression of cyclin E corresponded to up-regulation of cyclin D1 in the cells depleted of p120ctn but replete of either p120ctn-1A or 3A. In addition, when we knocked down cyclin D1 by siRNA in the cells depleted of p120ctn, the expression of cyclin E was not increased but slightly decreased, even though p120ctn isoform 1 or 3 was apparently restored by co-transfection of either isoform DNA plasmid. A similar phenomenon was observed when cyclin D1 was blocked by monoclonal antibody incubation (100 ng/ml, 48 h) in p120ctn reconstituted cells, implying a pivotal role of cyclin D1 in regulating cyclin E expression by either p120ctn isoform 1 or isoform 3. Furthermore, when translation of cyclin D1 mRNA in A549 was interrupted by siRNA, both transcript and protein levels of cyclin E were correspondingly down-regulated, suggesting p120ctn might not only stabilize cyclin E protein as previously reported but also promote transcription of cyclin E, presumably via an enhanced expression of cyclin D1.

The data in the present study demonstrated both p120ctn isoform 1 and 3 could up-regulate cyclin D1 expression, though it has not been entirely elucidated how the different p120ctn isoforms, particularly isoform 3, achieve this function. Our study showed that p120ctn isoform 1 could up-regulate the expression of β-catenin,especially increasing the active form of β-catenin, while isoform 3 could interact with Kaiso. Although p120ctn isoform 1 was overexpressed in A549 cells transfected with p120ctn-1A, the p120ctn-1/Kaiso complex remained undetected, implying a low level of p120ctn isoform 1 in A549 is not the cause of this undetectable protein complex. Thereby, questions are raised as to why isoform 1 could not interact with Kaiso and why isoform 3 could not up-regulate β-catenin. We hypothesized that the functional difference might be owing to the structural variations of these two isoforms at the N-terminus. The human CTNND1 gene comprises 21 exons and encodes potentially up to 48 protein isoforms due to multiple alternative inter- and intra-exonic splicing events [14]. Human isoforms, designated 1 to 4, differ from each other in the start codon used. Several domains have been identified in p120ctn, including the coiled coil domain found only in isoform 1. In fact, p120ctn isoform 1 is longer than p120ctn isoform 3 by 101 amino acid residues in the N-terminus [14]–[15]. In order to answer the two questions given above, we constructed two mutants of p120ctn isoform 1 with truncated peptide sequences at the N-terminus (M1, M2).

Our data suggest that, first, the deletion of the N-terminal 56-101 (N-56-101) amino acid residues in p120ctn isoform 3 could possibly explain its lack of regulatory effect on β-catenin, while p120ctn isoform 1, which has an intact N-terminal domain, showed a positive regulatory effect (Figure 6); secondly, the existence of N-1-55 amino acid residues, which form a coiled coil domain, in p120ctn isoform 1 may dominantly prevent it from binding to Kaiso (Figure S9), though N-56-101 amino acid residues may play a minor role in their interaction.

In the current study, we demonstrated an increased active form of β-catenin without change at the transcript level in the cells with overexpression of p120ctn isoform 1A. The finding is consistent with what has been previously described in the literature. It has been reported that in vivo conditions, p120ctn isoform 1 directly or indirectly interacts with some of the key proteins known to regulate β-catenin stability such as Axin and GSK-3, in addition, it shares with β-catenin the same regulatory mechanisms of metabolic stability [16]. Overexpression of p120ctn isoform 1 may occupy more binding sites on the destruction-complexes which contain Axin and GSK-3β and, as a result, cause reduced degradation of β-catenin by replacing it from the destruction-complexes. The binding sites of p120ctn with GSK-3β and CK1α are located in the N-terminal domain of p120ctn, and, thus, the p120ctn isoform 1 protein, which has the complete N-terminal domain, would be subjected to regulation by the destruction-complex [16]. The findings in this study suggest that the more specific domain, precisely the N-56-101 amino acid residues, may be involved in degradation of p120ctn isoform 1. In addition, p120ctn-1 M1 constructed in this study is structurally the same as p120ctn isoform 2 and also showed up-regulation of β-catenin (Figure 6), suggesting that p120ctn isoform 2 might also combine with GSK-3β and CK1α and be regulated by the destruction-complex.

The coiled coil domain, located in the N-1-55 amino acid residues of p120ctn isoform 1, may participate in protein–protein interactions [17]. Our study results suggest that the coiled coil domain might prevent p120ctn isoform 1 from binding to Kaiso (Figure S9). Theoretically, the coiled coil domain probably generates a specific conformation of p120ctn isoform 1, and thereby conceals its binding site with Kaiso in the Armadillo sequence. We noted that M2 still has some remaining binding affinity with Kaiso (Figure S9), while p120ctn-1/Kaiso complex is undetectable by all the methods used in our study. This phenomenon indicates that the N-56-101 amino acid residues also have their role in the protein-protein interaction. These amino acid residues may increase the distance of the coiled coil domain to the Arms domain and lead to a more subtle conformation change of p120ctn isoform 1, resulting in an alteration of its binding affinity with Kaiso. In addition, we noted that, M2 still has some remaining binding affinity with Kaiso, but could not up-regulate the expression of cyclin D1 (Figure 6). The binding affinity of M2 with Kaiso may be not strong enough to take Kaiso away from the promoter of cyclin D1 and transport it out of nucleus through the nuclear pore complex.

In addition, the two lung cancer cell lines show different subcellular distribution of Kaiso, mainly localized in the nucleus of A549 but primarily distributed in the cytoplasm of SPC. Western blot analysis showed that p120ctn isoform 3 in the SPC cells was 6 times more than that in the A549 cells, implying that p120ctn isoform 3 may have a role in the nuclear export of Kaiso. The data in this study suggest this different distribution of Kaiso may be related to the different amount of p120ctn isoform 3 between these two cell lines. Restoration of p120ctn-3A could decrease Kaiso in the nucleus, and transfection of NLS-p120ctn-3A plasmid or suppression of the nuclear export of p120ctn-3A by LMB inhibited Kaiso export from the nucleus. While both p120ctn and Kaiso have NLS, which guides proteins into the nucleus through the importin α/β pathway [18]–[19], only p120ctn has NES, which facilitates the nuclear export of proteins via CRM-1 pathway [20]–[21]. However, it is currently unclear how Kaiso is transported from nucleus to cytoplasm. Based on our data, we speculated that the nuclear export of Kaiso is likely to be p120ctn-3-dependent. Conversely, because it failed to combine with Kaiso, p120ctn isoform 1 could not regulate the nucleocytoplasmic shuttle of Kaiso as did p120ctn isoform 3.

Of note, in the present study, we have shown that transient transfection of p120ctn-1A or p120ctn-3A could not change the protein expression of Kaiso; however, the change of Kaiso protein expression has been detected after stable knockdown of p120ctn or stable transfection of p120ctn-1A or p120ctn-3A in our previous investigation [5]. Cells with stable knockdown or over-expression of p120ctn may reach a new biological equilibrium to cope with an altered signal profile and/or a new cell proliferation rate. The change may take some time to equilibrate, and thus, may not reach a detectable level after transient transfection of p120ctn in the present study. Nonetheless, these contrasting results based on stable and transient modulation of p120ctn and subsequent dynamic changes of kaiso should be noted in the future investigations.

In conclusion, both p120ctn isoform 1 and 3 promote cell proliferation and cell cycle progression. Nonetheless, they seem to achieve the effect via different pathways, even though both pathways converge to up-regulate cyclin D1. While p120ctn isoform 3 up-regulates cyclin D1 via binding to Kaiso, facilitating its export from the nucleus and, thus, relieving its transcriptional repression on the cyclin D1 gene, p120ctn isoform 1 accomplishes this function via up-regulating β-catenin at the protein level, likely stabilizing its active form. In addition, our data provide for the first time an insight into the different functions of p120ctn isoforms, which might be due to the variations of their N-terminal structure, though a complete elucidation of their roles in the canonical Wnt pathway as well as in cell cycle control remains to be further investigated. We hope, that with additional elucidation of the Wnt pathway and the roles of p120ctn isoforms in carcinogenesis, particularly in the development of lung cancer, certain pharmacologic or antibody-based therapeutic agents could be designed to target the key mediators in the Wnt pathway in order to circumvent the side effects of conventional chemotherapy currently used and, ultimately, improve the survival and quality of life for lung cancer patients.

## Materials and Methods

### Cell culture

Human lung adenocarcinoma cell lines A549 and SPC were obtained from the American Type Culture Collection (Manassas, VA, USA). The cells were cultured in RPMI 1640 medium (Invitrogen, Carlsbad, CA, USA) containing 10% fetal calf serum (Invitrogen), 100 IU/ml penicillin (Sigma, St. Louis, MO, USA) and 100 µg/ml streptomycin (Sigma).

### Plasmid construction and transfection

For the production of the p120ctn-siRNA (GeneBank#: 001331) plasmids used in the experiments, sense and anti-sense oligonucleotides were annealed and inserted between BamHI and HindIII sites of the pGCsi vector (Shanghai GeneChem Co. Ltd., Shanghai, China). The sequences of the two double-stranded oligonucleotides were as follows:

A: 5′-GGATCACAGTCACCTTCTA-3′;

5′-TAGAAGGTGACTGTGATCC-3′

B: 5′-GCACTTGTATTACAGACAA-3′;

5′-TTGTCTGTAATACAAGTGC-3′

A single cell clone was selected for adequate efficacy and specificity. To construct p120ctn- 1A/3A nuclear target localization plasmids, full-length DNA sequences of murine p120ctn isoforms 1A and 3A were generated by polymerase chain reaction (PCR) using RacCMV-Kpnl/p120-1/3A (a gift from AB. Reynolds, Vanderbilt University Medical School, Nashville, TN) as a template. Then, the PCR products were subcloned into the nucleus- localization expression vector pCMV/myc/nuc (invitrogen, Carlsbad, CA) to generate pCMV/p120ctn-1A/nuc (NLS-p120-1A) and pCMV/p120ctn-3A/nuc (NLS-p120-3A) plasmids. pBluescript-Kaiso plasmid was a gift from Juliet M. Daniel, McMaster University, Hamilton, Canada. Two deletion mutants of p120ctn-1 M1 and M2, which lack N-1-55 amino acid residues and N-56-101 amino acid residues respectively (Figure S10), were constructed by TaKaRa (TaKaRa, DaLian, China).

Three Kaiso shRNA plasmids (RHS1764-9214280, RHS1764-9216302, and RHS1764-9692262) and a non-silencing pSM2 shRNA control plasmid (RHS1707) were purchased from the Open Biosystems Company. β-catenin, cyclin D1 and cyclin E siRNA oligonucleotides were purchased from Santa Cruz Biotechnology Inc, CA, USA. The plasmids were transfected with Lipofectamine 2000 (invitrogen, Carlsbad, CA) or Attractene Transfection Reagent (QIAGEN GmbH, Hilden, Germany) into cells to accomplish the transient and stable transfection according to the manufacturers' protocols. The empty plasmid was used as a negative control. Selection was accomplished with G418 (Invitrogen). Drug-resistant cells were tested for the absence of p120ctn expression by Western blot and reverse transcription-PCR. Leptomycin B (LMB, sigma- aldrich, USA, final concentration, 50 nM 18 h) was used to block the nuclear export of p120ctn isoform 3.

### 3-(4,5-Dimethylthiazol-2-yl)-2,5-Diphenyltetrazolium Bromide (MTT) Assay

Cell proliferation was evaluated each day for four days after the MTT treatment. The absorbance, which is directly correlated with the number of viable cells in the culture, was measured at 550 nm using a microplate reader (Model 550, Bio-Rad, Hercules, CA, USA). A blank with dimethyl sulfoxide (DMSO) alone was measured and the value was subtracted from all the absorbance for cell culture specimens.

### Flow cytometry (FCM)

After 48 hours of culture, cells from each experimental group were collected and digested with trypsin and fixed with 75% ice-cold ethanol at 4°C overnight. Cells (1×10^6^) were centrifuged at 1500 rpm for 5 min, and the pellets were resuspended with 50 µg/ml propidium iodide (Sigma) for 45 min in the dark before analysis. The percentages of cells in different phases of cell cycle were determined using a FACSCalibur Flow Cytometer with CellQuest 3.0 software (BD Biosciences). Experiments were performed in triplicate.

### RT-PCR

RT-PCR was performed with the RNA PCR Kit (AMV) Version 3.0, according to the manufacturer's instructions.

### Western Blot

Each extracted protein sample (50 µg) was separated by SDS-PAGE. After transferring to polyvinylidene fluoride (PVDF) membrane, the membrane was incubated overnight at 4°C with either the mouse monoclonal antibody against p120ctn (1∶500, BD Tranduction Laboratories, USA), Kaiso(1∶500, Upstate Biotechnology, LakePlacid, NY, USA), β-catenin (1∶1000, BD Tranduction Laboratories, USA), Active β-catenin(1∶400 Santa Cruz Biotechnology Inc, CA, USA), cyclin D1(1∶200, Santa Cruz Biotechnology Inc, CA, USA), cyclin E(1∶500, Upstate Biotechnology) or β-actin(1∶500, Santa Cruz Biotechnology Inc). After incubation with peroxidase-coupled anti-mouse IgG at 37°C for 2 hours, the protein bands were visualized using ECL (Pierce, Rockford, IL, USA) and detected using the BioImaging Systems (UVP Inc.). The relative protein levels were calculated in reference to the amount of β-actin protein.

### Chromatin immunoprecipitation assay (ChIP)

Chromatin immunoprecipitation assay kit (Upstate Biotechnology, Lake Placid, NY) was used according to the instructions recommended by the manufacturer. Briefly, cells were plated at a density of 10^6^ to 10^7^ per 100-mm dish and cultured for 24 hours followed by 12 hours of culture with 500 nmol/l trichostatin A. Subsequently, chromatin was solubilized and subjected to sonication to obtain DNA fragments with an average size of 200 to 1,000 bp. Chromatin immunoprecipitation was carried out by incubation with 4 µg anti-acetyl histone H3 antibody (Upstate Biotechnology) or without antibody as a control. Ten percent of precleared lysate was saved for each sample to determine the amount of input chromatin. Immunoprecipitated DNA was used as a template for PCR of the cyclin D1 promoter. The cyclin D1 promoter sequence (GenBank: AY439218.1) was identified in GENEBANK, and PCR primers were designed spanning the KBS element on the promoter region (TAKARA company was commissioned). The primers used are listed in Table 1.

**Table 1:** RT-PCR primer sequences and amplification conditions.

Primer sequences (5′→3′)		Length	PCR setting		
		(bp)	Denature	Annealing	Extension
β-catenin	5′-GCCAAGTGGGTGGTATAGAG-3′	330	95°C	53°C 40 s	72°C
	5′-GCTGGGTATCCTGATGTGC-3′		40 s	40 cycles	40 s
Kaiso	5′-TGCCTATTATAACAGAGTCTTT-3′	116	95°C	55°C 40 s	72°C
	5′-AGTAGGTGTGATATTTGTTAAAG-3′		40 s	40 cycles	40 s
cyclin D1	5′-CCCGATGCCAACCYCCTCAA-3′	500	95°C	58°C 40 s	72°C
	5′-TGGCACAGAGGGCAACGAAG-3′		40 s	40 cycles	40 s
cyclin E	5′-CTGGATGTTGACTGCCTTGA-3′	359	95°C	55°C 40 s	72°C
	5′-CCGCTGCTCTGCTTCTTAC-3′		40 s	40 cycles	40 s
cyclin D1	5′-CCCTCTCATGTAACCACGAA-3′	179	95°C	58°C 40 s	72°C
promoter	5′-TGGTTTTGTTGGGGGTGTAG-3′		40 s	40 cycles	40 s
β-actin	5′-AGAGCTACGAGCTGCCTGAC-3′	308	95°C	55°C 40 s	72°C
	5′-AGTACTTGCGCTCAGGAGGA-3′		40 s	40 cycles	40 s
GAPDH	5′-AAGGCTGGGGCTCATTTGCAG-3′	166	95°C	58°C 40 s	72°C
	5′-GGCCAGGGGTGCTAAGCAGTT-3′		40 s	40 cycles	40 s

### Immunofluorescent staining

Cells grown on glass coverslips were fixed with ice-cold 100% methanol for 15 minutes at −20°C, followed by permeabilization with 0.2% Triton X-100. Kaiso was detected using goat polyclonal antibodies (Santa Cruz Biotechnology Inc, CA, USA) and p120ctn was detected using mouse monoclonal antibodies. Primary antibodies were applied overnight at 4°C followed by incubation with secondary antibody conjugated to rhodamine/fluorescein isothiocyanate (FITC). The nuclei were counterstained with propidium iodide/4, 6 diamidino-2-phenylindole. The cells were examined with an Olympus IX51 fluorescent microscope (Olympus, Tokyo, Japan), and images were captured with a CoolPIX 5400 camera (Nikon, Japan).

### Immunoprecipitation

Cells were washed twice with 5 ml of PBS followed by incubation on ice with lysis buffer containing 0.5% NP-40, 50 mM Tris, 150 Mm NaCl, 1 mM phenylmethylsulfonyl fluoride, 5 mg/ml leupeptin, 2 mg/ml aprotinin, 1 mM sodium orthovanadate, and 1 mM EDTA for 5 minutes. Cells were harvested from the plates, and transferred to a 1.5 ml tube. The lysate was centrifuged at 16,000 g for 5 minutes at 4°C and the supernatant was transferred to a new tube. Lysates were quantified by Bradford assay and equal amounts of total protein were used for immunoprecipitation with the anti-p120ctn, anti-p120ctn-1, 2 (6H11, Upstate Biotechnology, LakePlacid, NY, USA), anti-GFP (Santa Cruz Biotechnology Inc, CA, USA) or anti-kaiso mAb. The immunocomplexes were then subjected to SDS-PAGE.

### Statistical Analysis

All data were expressed as means ± standard deviation (S.D.) for in vitro experiments performed at least 3 times, and the one-way analysis of variance followed by a least significant different test (LSD) for multiple comparisons was used for statistical analysis via SPSS 13.0 for Windows (SPSS Inc., Chicago, IL, USA). P values less than 0.05 were considered statistically significant.

## Supporting Information

Figure S1 The expression and localization of p120ctn and E-cadherin were detected in A549 and SPC cells. (**A**) Western blot showed p120ctn-1/3 and E-cadherin expression in A549 and SPC cells *in vivo* and they are higher in SPC cells. (**B**) p120ctn and E-cadherin were mainly localized at the cytoplasm of A549 and SPC cells. No visibly membranous signal was observed in these two cell lines.
(TIF)

Figure S2 p120ctn-1 and 3 regulate the transcription of cyclin D1 and cyclin E. (**A**) The results of RT-PCR showed that the mRNA of cyclin D1 (*, *p* = 0.004) and cyclin E (*, *p* = 0.003) were significantly decreased in A549 cells with knocked down p120ctn. (**B**) The mRNA of cyclin D1 (*, *p*<0.001; **, *p* = 0.002) and cyclin E (*, *p*<0.001; **, *p* = 0.002) were significantly recovered after p120ctn-1A or 3A plasmids were transfected in A549 cells depleted of p120ctn.
(TIF)

Figure S3 p120ctn-1 and 3 regulate the expression of cyclin D1 and cyclin E, affect the cell proliferation and cell cycle in SPC cells. (**A** and **B**) Western blot and RT-PCR showed that the expression of p120ctn, cyclin D1 (protein, +, *p* = 0.000, mRNA, +, *p* = 0.000) and cyclin E (protein, +, *p* = 0.011, mRNA, +, *p* = 0.000) in SPC-K2 cells were significantly lower than those in SPC cells. When we restored the expression of p120ctn-1A and 3A, the protein and mRNA expression of cyclin D1 (protein, *, *p* = 0.001; **, *p* = 0.003, mRNA, *, *p* = 0.000; **, *p* = 0.000) and cyclin E (protein, *, *p* = 0.003; **, *p* = 0.002, mRNA, *, *p* = 0.000; **, *p* = 0.000) significantly recovered, and the effect of p120ctn-1A was stronger than p120ctn-3A (*p*<0.05). (**C**) The result of MTT assay showed that transfection of p120ctn-1A or p120ctn-3A in SPC-K2 cells significantly increased cell proliferation 48 hours later (*p*<0.01), and the effect of p120ctn-1A was stronger than p120ctn-3A (*p*<0.05). The G1 phase cells of SPC-K2 was more than SPC (+, *p*<0.01), and S phase cells was less (+, *p*<0.01). When we transfected p120ctn-1A and 3A 48 h later, the G1 phase cells ratio of SPC-K2 decreased significantly (*, *p*<0.01, **, *p*<0.01), and S phase cells ratio significantly increased (*, *p*<0.01, **, *p*<0.01).
(TIF)

Figure S4 Cyclin D1 could regulate cyclin E transcription. (**A**) Cyclin D1 depletion by siRNA in A549 cells led to reduced transcription of cyclin E, but conversely, the mRNA of cyclin D1 was not significantly changed (*p* = 0.664) in cells with knocked down cyclin E by siRNA. (**B**) After co-transfection of siRNA-cyclin D1 with p120ctn isoform 1 or 3 in the cells depleted of p120ctn for 48 hours, the mRNA of cyclin E was not increased. The comparison is made to the control group.
(TIF)

Figure S5 In p120ctn knocked down A549 cells, which were transfected with p120ctn-1A or 3A later, incubating with monoclonal cyclin D1 antibody (100 ng/ml) for 48 hs resulted in reduced cyclin E expression, both at protein and mRNA levels.
(TIF)

Figure S6 The result of confocal immunofluorescence showed that overexpression of p120ctn-1A significantly increased β-catenin in cell nucleus/cytoplasm. β-catenin was localized both in the nucleus and cytoplasm of A549 and SPC cells. With p120ctn depleted, β-catenin was significantly reduced. Overexpression of p120ctn-1A significantly rebounded β-catenin. However, with the transfection of p120ctn-3A, the expression and localization of β-catenin did not change significantly.
(TIF)

Figure S7 Kaiso regulates the transcription of cyclin D1 and cyclin E. (**A**) RT-PCR analyses show the increased mRNA of Kaiso in A549 cells transfected with Kaiso cDNA. Kaiso overexpression remarkably down-regulated the transcription of cyclin D1 (*p* = 0.000) and cyclin E (*p* = 0.002). (**B**) RT-PCR analyses show reduced mRNA of Kaiso in SPC cells transfected with Kaiso siRNA. Kaiso interference significantly increased the transcription of cyclin D1 (*p* = 0.004) and cyclin E (*p* = 0.003). All the comparisons are made to the group of cells transfected with vector alone.
(TIF)

Figure S8 p120ctn-3 could regulate the subcellular localization of Kaiso. (**A**) Kaiso is mainly localized in the cytoplasm of SPC cells, which express relatively high levels of p120ctn, whereas, Kaiso is mainly localized in the nucleus in p120ctn depleted SPC cell lines. Kaiso is still predominantly localized in the nucleus after restoration of p120ctn-1A in SPC-K2 cells. Kaiso came back to the cytoplasm after the restoration of p120ctn-3A. (**B**) Kaiso is mainly distributed in the nucleus of A549 cell lines, which express low levels of p120ctn. After transfected with p120ctn-1A, subcellular localization of Kaiso did not change significantly. Kaiso was mainly localized in the cytoplasm of cells transfected with p120ctn-3A. Furthermore, LMB was used to block the nuclear export of p120ctn in cells transfected with p120ctn-1A and p120ctn-3A. Kaiso is mainly localized in the nucleus with LMB incubation. A549 cells transfected with NLS-p120ctn-1A and 3A plasmids, still showed Kaiso localized in the nucleus.
(TIF)

Figure S9 Co-immunoprecipitation was carried out following transfection of M1 or M2 with sufficient protein and equivalent GFP monoclonal antibody. The GFP monoclonal antibody could effectively precipitate exogenous deletion mutants of p120ctn-1 and Kaiso protein, and the binding affinity of M1 seems to be significantly stronger than M2, implying the coiled coil domain, located in the N-1-55 amino acid residues of p120ctn isoform 1, might prevent p120ctn isoform 1 from binding to Kaiso.
(TIF)

Figure S10 Two deletion mutants of p120ctn-1 M1 and M2 lack N-1-55 amino acid residues and N-56-101 amino acid residues respectively.
(TIF)
