# Supplementary figures and images for: P120-Catenin Isoforms 1 and 3 Regulate Proliferation and Cell Cycle of Lung Cancer Cells via β-Catenin and Kaiso Respectively
Source: PLoS One. 2012 Jan 20;7(1):e30303. doi: 10.1371/journal.pone.0030303 (PMC3262806; doi:10.1371/journal.pone.0030303)

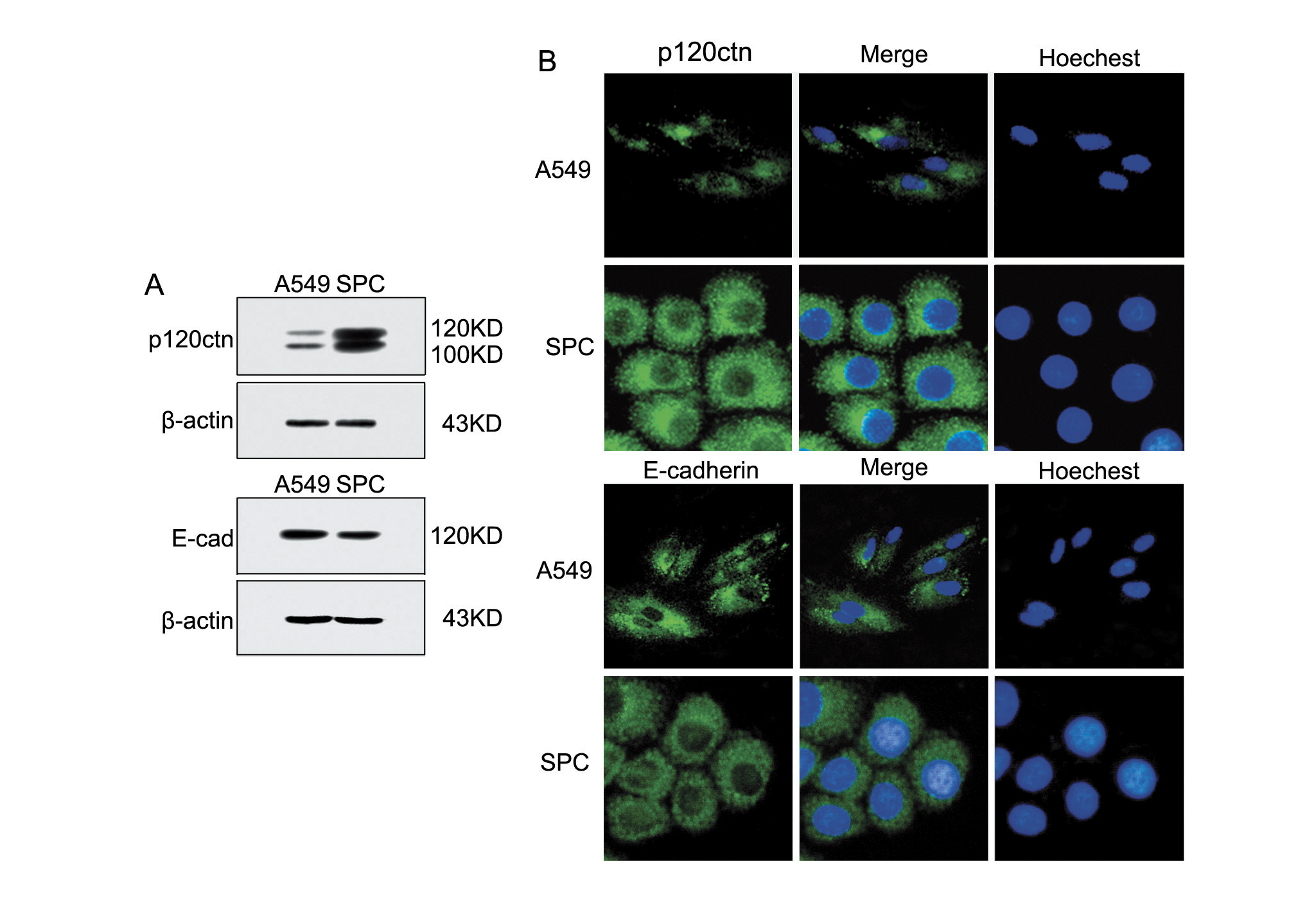

Supplement: Figure S1 — The expression and localization of p120ctn and E-cadherin were detected in A549 and SPC cells. (A) Western blot showed p120ctn-1/3 and E-cadherin expression in A549 and SPC cells in vivo and they are higher in SPC cells. (B) p120ctn and E-cadherin were mainly localized at the cytoplasm of A549 and SPC cells. No visibly membranous signal was observed in these two cell lines. (TIF) [file pone.0030303.s001.tif]

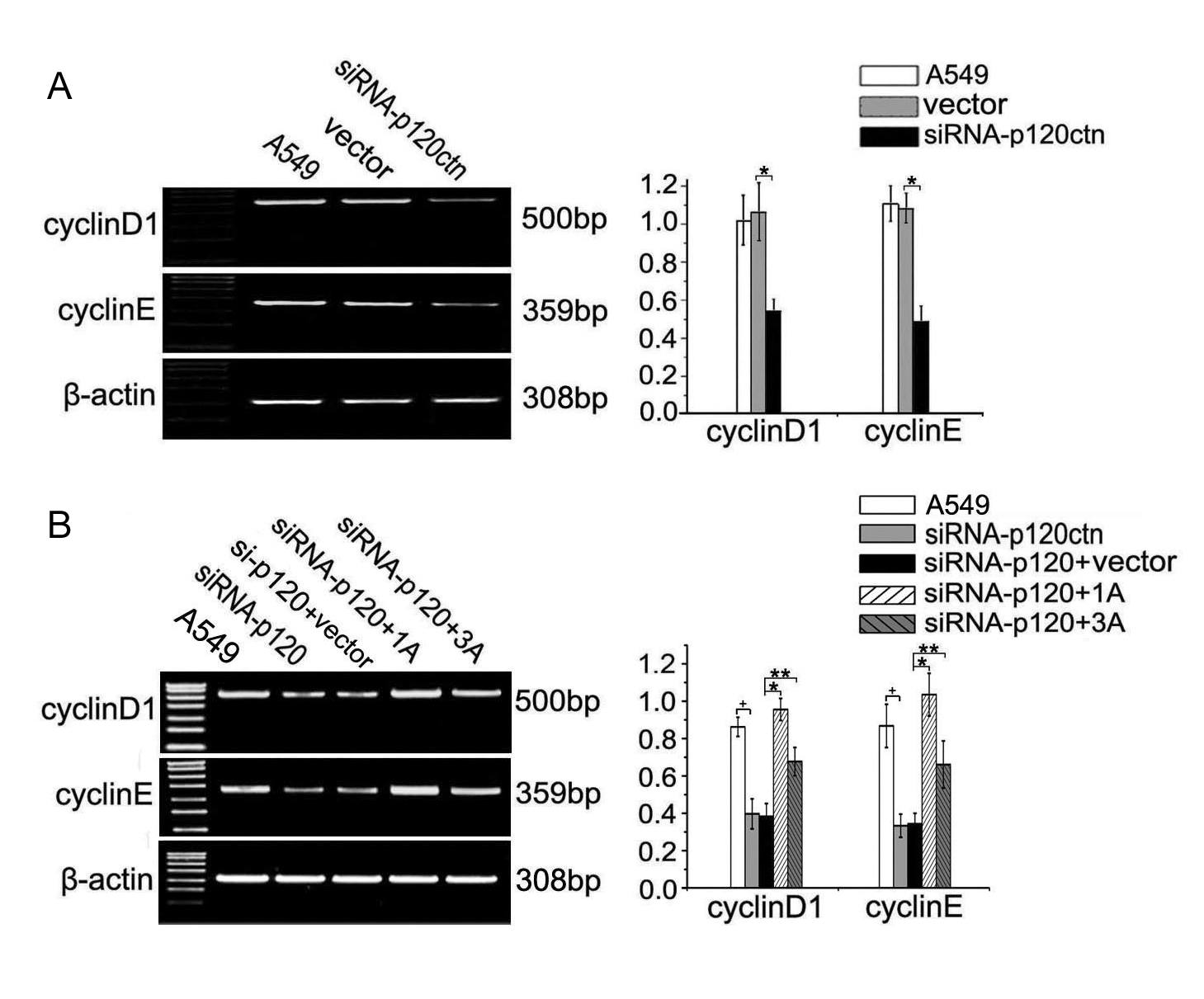

Supplement: Figure S2 — p120ctn-1 and 3 regulate the transcription of cyclin D1 and cyclin E. (A) The results of RT-PCR showed that the mRNA of cyclin D1 (*, p = 0.004) and cyclin E (*, p = 0.003) were significantly decreased in A549 cells with knocked down p120ctn. (B) The mRNA of cyclin D1 (*, p<0.001; **, p = 0.002) and cyclin E (*, p<0.001; **, p = 0.002) were significantly recovered after p120ctn-1A or 3A plasmids were transfected in A549 cells depleted of p120ctn. (TIF) [file pone.0030303.s002.tif]

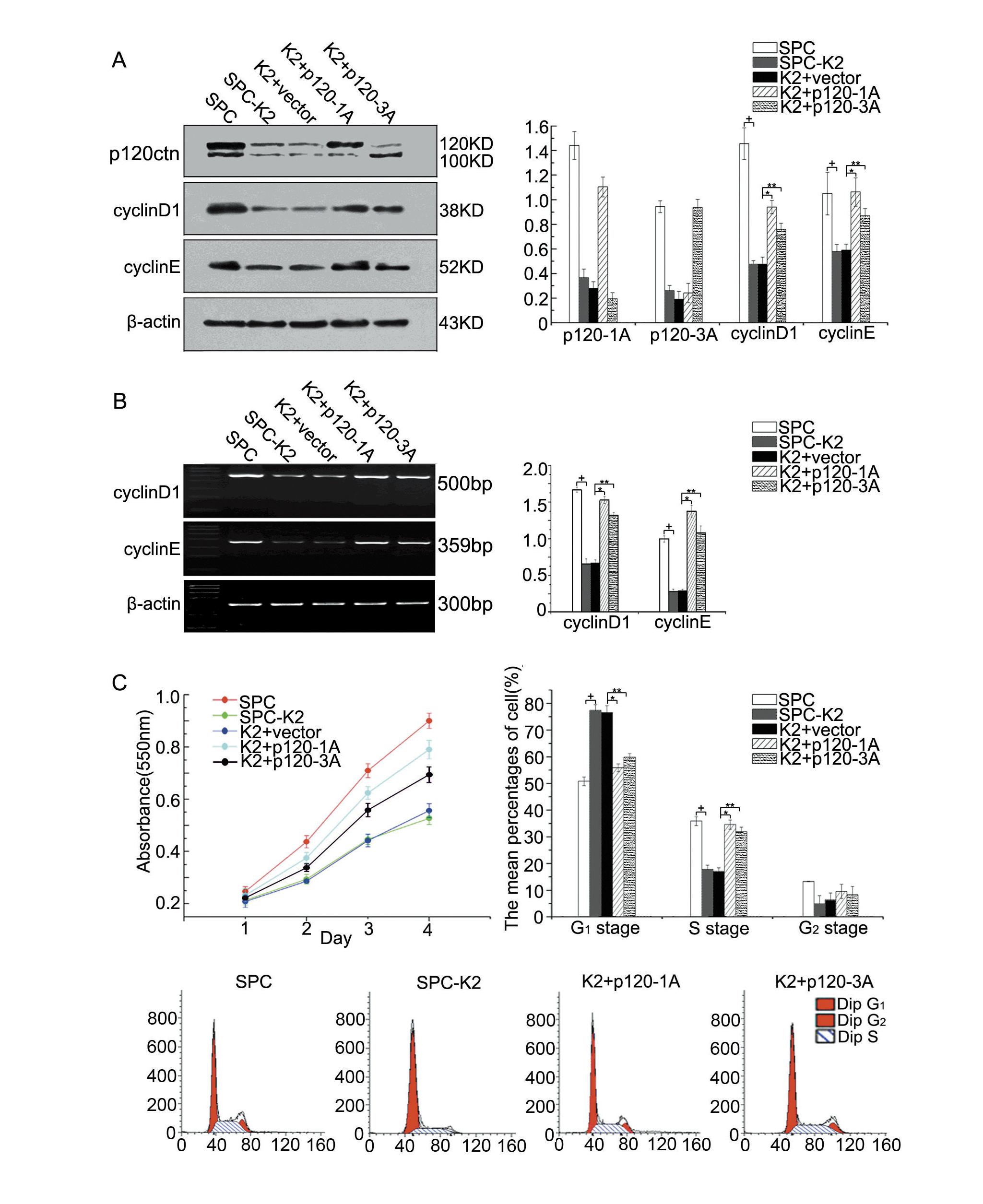

Supplement: Figure S3 — p120ctn-1 and 3 regulate the expression of cyclin D1 and cyclin E, affect the cell proliferation and cell cycle in SPC cells. (A and B) Western blot and RT-PCR showed that the expression of p120ctn, cyclin D1 (protein, +, p = 0.000, mRNA, +, p = 0.000) and cyclin E (protein, +, p = 0.011, mRNA, +, p = 0.000) in SPC-K2 cells were significantly lower than those in SPC cells. When we restored the expression of p120ctn-1A and 3A, the protein and mRNA expression of cyclin D1 (protein, *, p = 0.001; **, p = 0.003, mRNA, *, p = 0.000; **, p = 0.000) and cyclin E (protein, *, p = 0.003; **, p = 0.002, mRNA, *, p = 0.000; **, p = 0.000) significantly recovered, and the effect of p120ctn-1A was stronger than p120ctn-3A (p<0.05). (C) The result of MTT assay showed that transfection of p120ctn-1A or p120ctn-3A in SPC-K2 cells significantly increased cell proliferation 48 hours later (p<0.01), and the effect of p120ctn-1A was stronger than p120ctn-3A (p<0.05). The G1 phase cells of SPC-K2 was more than SPC (+, p<0.01), and S phase cells was less (+, p<0.01). When we transfected p120ctn-1A and 3A 48 h later, the G1 phase cells ratio of SPC-K2 decreased significantly (*, p<0.01, **, p<0.01), and S phase cells ratio significantly increased (*, p<0.01, **, p<0.01). (TIF) [file pone.0030303.s003.tif]

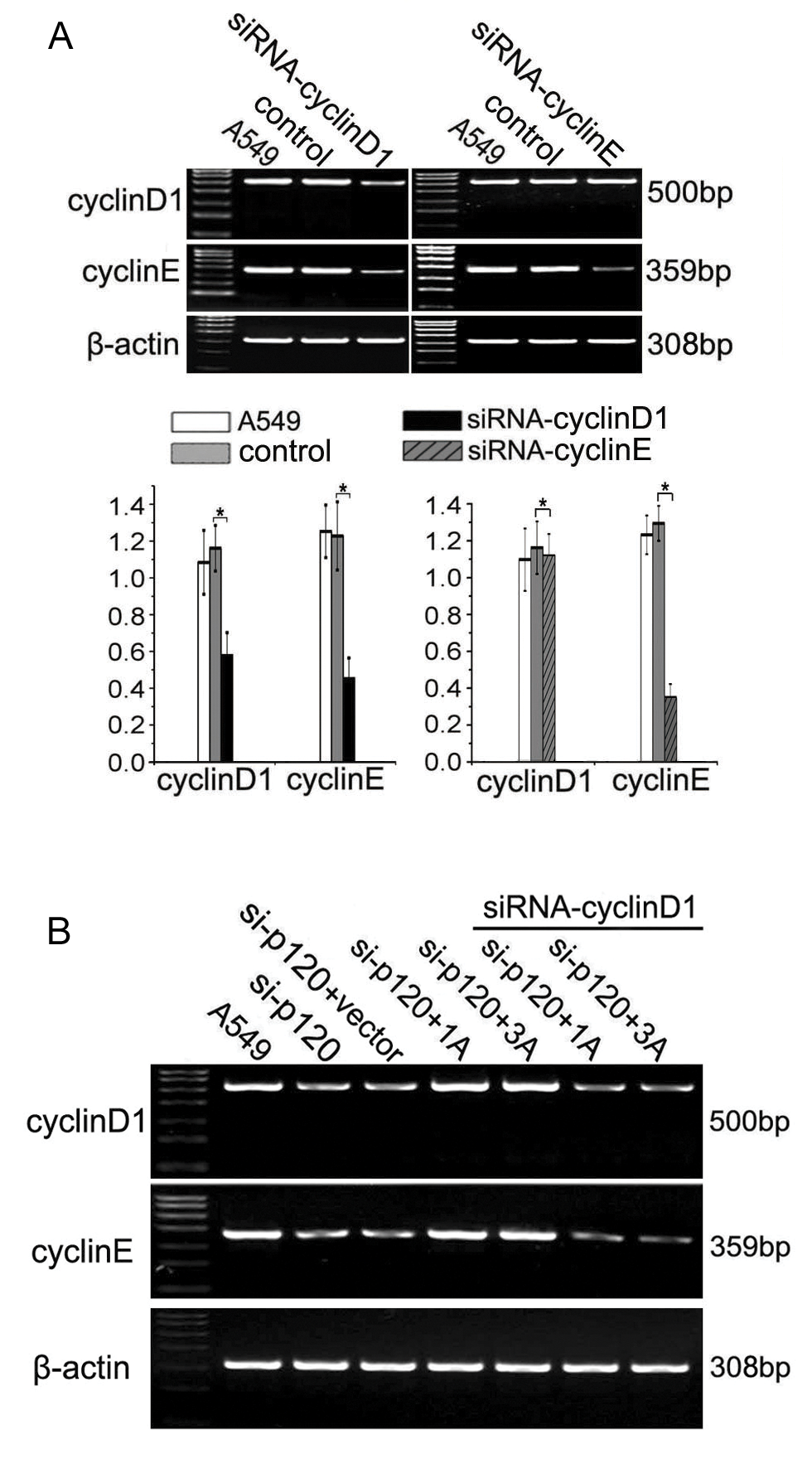

Supplement: Figure S4 — Cyclin D1 could regulate cyclin E transcription. (A) Cyclin D1 depletion by siRNA in A549 cells led to reduced transcription of cyclin E, but conversely, the mRNA of cyclin D1 was not significantly changed (p = 0.664) in cells with knocked down cyclin E by siRNA. (B) After co-transfection of siRNA-cyclin D1 with p120ctn isoform 1 or 3 in the cells depleted of p120ctn for 48 hours, the mRNA of cyclin E was not increased. The comparison is made to the control group. (TIF) [file pone.0030303.s004.tif]

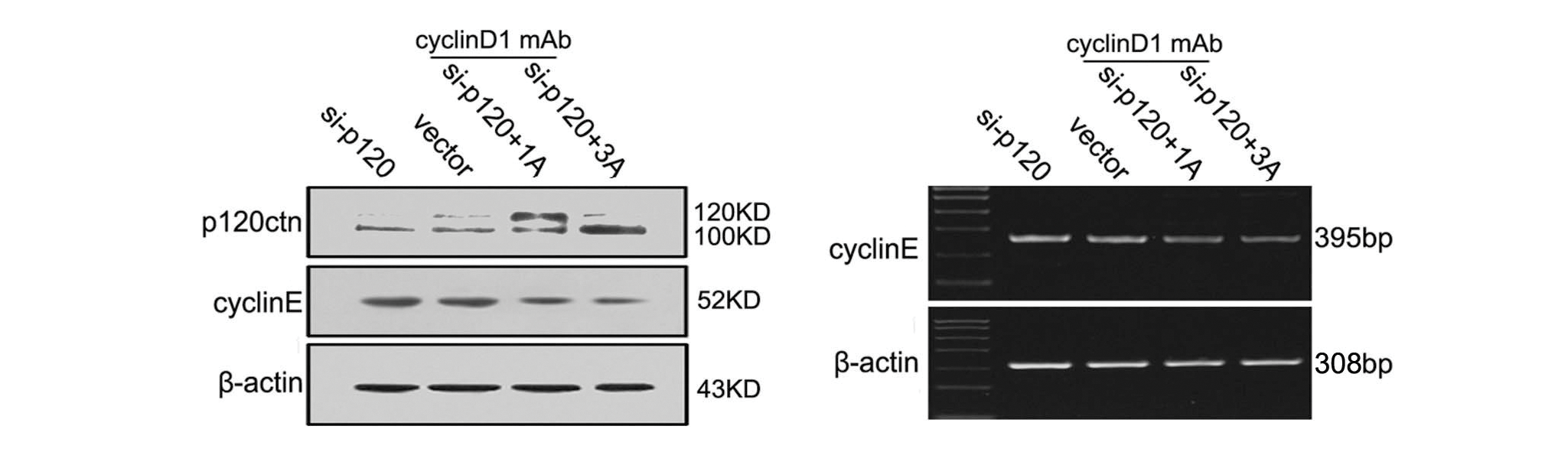

Supplement: Figure S5 — In p120ctn knocked down A549 cells, which were transfected with p120ctn-1A or 3A later, incubating with monoclonal cyclin D1 antibody (100 ng/ml) for 48 hs resulted in reduced cyclin E expression, both at protein and mRNA levels. (TIF) [file pone.0030303.s005.tif]

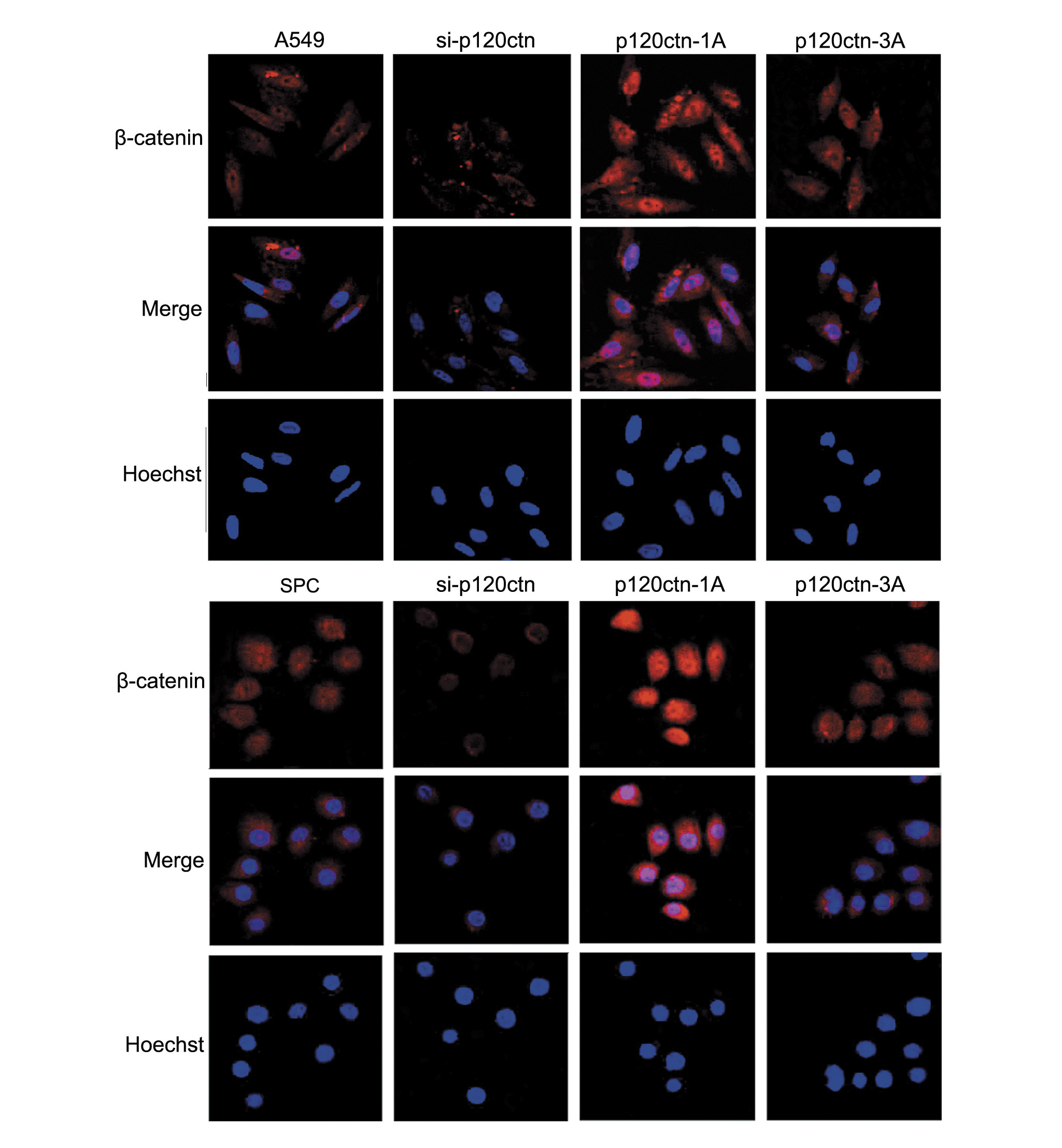

Supplement: Figure S6 — The result of confocal immunofluorescence showed that overexpression of p120ctn-1A significantly increased β-catenin in cell nucleus/cytoplasm. β-catenin was localized both in the nucleus and cytoplasm of A549 and SPC cells. With p120ctn depleted, β-catenin was significantly reduced. Overexpression of p120ctn-1A significantly rebounded β-catenin. However, with the transfection of p120ctn-3A, the expression and localization of β-catenin did not change significantly. (TIF) [file pone.0030303.s006.tif]

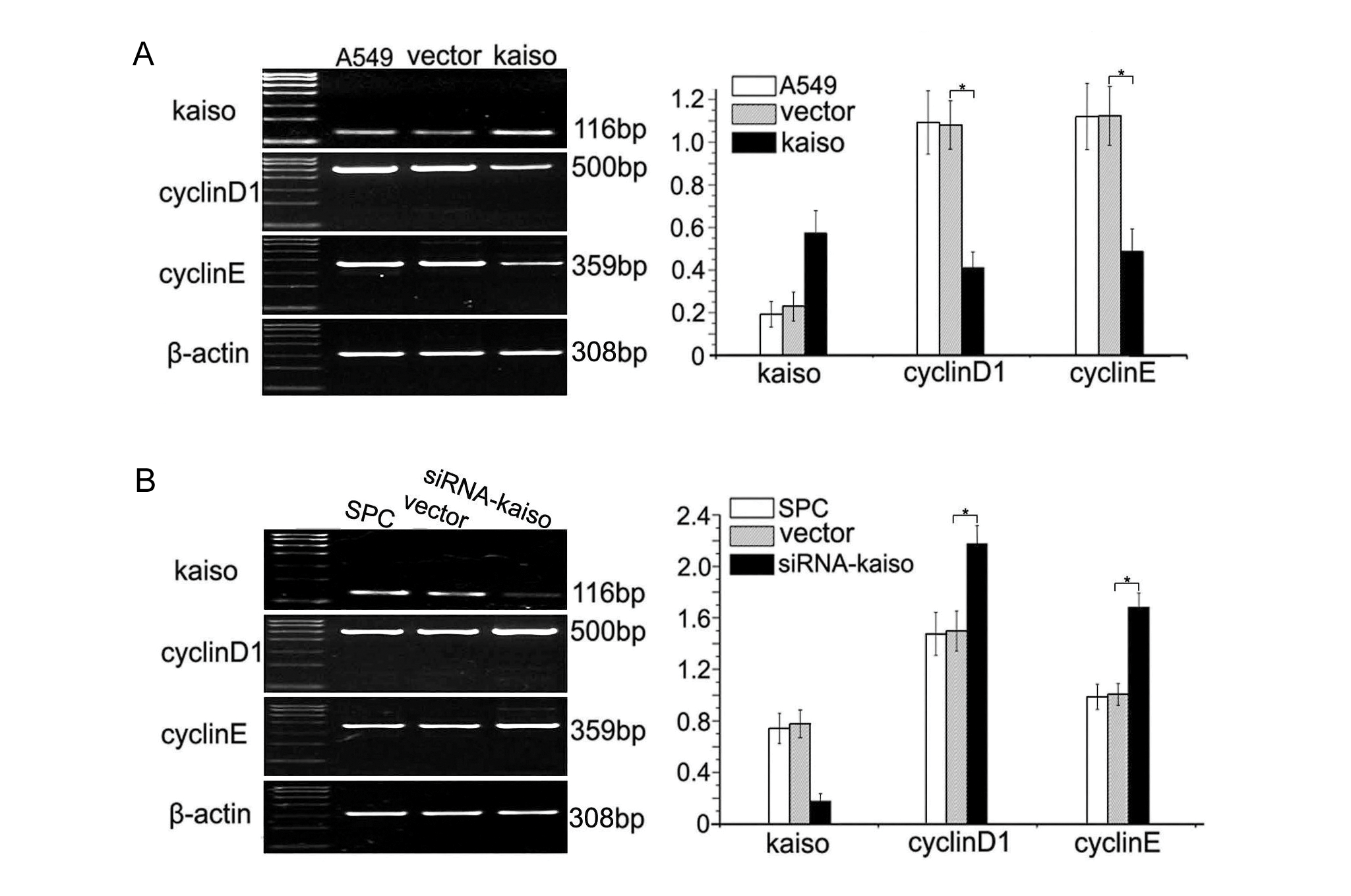

Supplement: Figure S7 — Kaiso regulates the transcription of cyclin D1 and cyclin E. (A) RT-PCR analyses show the increased mRNA of Kaiso in A549 cells transfected with Kaiso cDNA. Kaiso overexpression remarkably down-regulated the transcription of cyclin D1 (p = 0.000) and cyclin E (p = 0.002). (B) RT-PCR analyses show reduced mRNA of Kaiso in SPC cells transfected with Kaiso siRNA. Kaiso interference significantly increased the transcription of cyclin D1 (p = 0.004) and cyclin E (p = 0.003). All the comparisons are made to the group of cells transfected with vector alone. (TIF) [file pone.0030303.s007.tif]

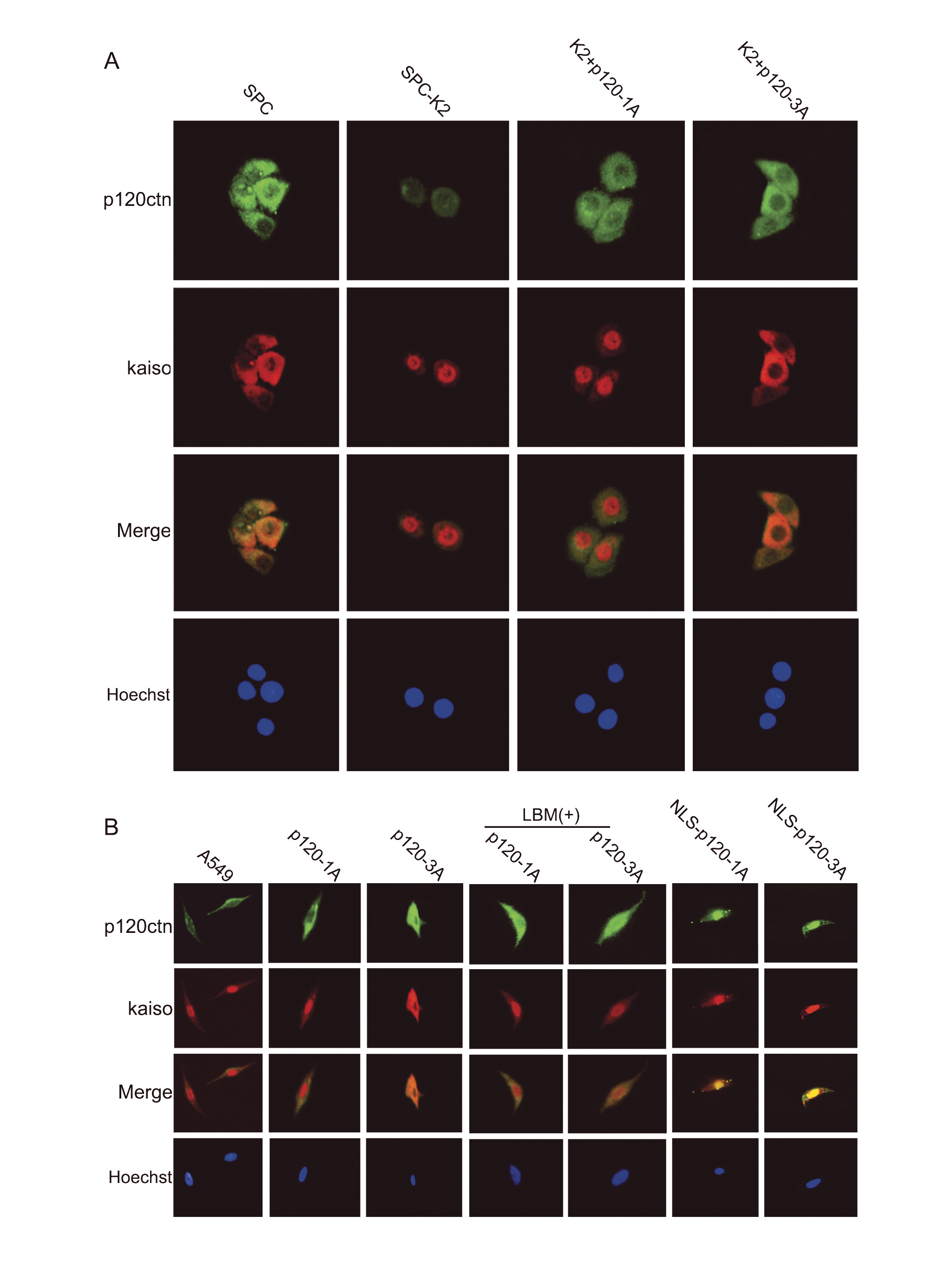

Supplement: Figure S8 — p120ctn-3 could regulate the subcellular localization of Kaiso. (A) Kaiso is mainly localized in the cytoplasm of SPC cells, which express relatively high levels of p120ctn, whereas, Kaiso is mainly localized in the nucleus in p120ctn depleted SPC cell lines. Kaiso is still predominantly localized in the nucleus after restoration of p120ctn-1A in SPC-K2 cells. Kaiso came back to the cytoplasm after the restoration of p120ctn-3A. (B) Kaiso is mainly distributed in the nucleus of A549 cell lines, which express low levels of p120ctn. After transfected with p120ctn-1A, subcellular localization of Kaiso did not change significantly. Kaiso was mainly localized in the cytoplasm of cells transfected with p120ctn-3A. Furthermore, LMB was used to block the nuclear export of p120ctn in cells transfected with p120ctn-1A and p120ctn-3A. Kaiso is mainly localized in the nucleus with LMB incubation. A549 cells transfected with NLS-p120ctn-1A and 3A plasmids, still showed Kaiso localized in the nucleus. (TIF) [file pone.0030303.s008.tif]

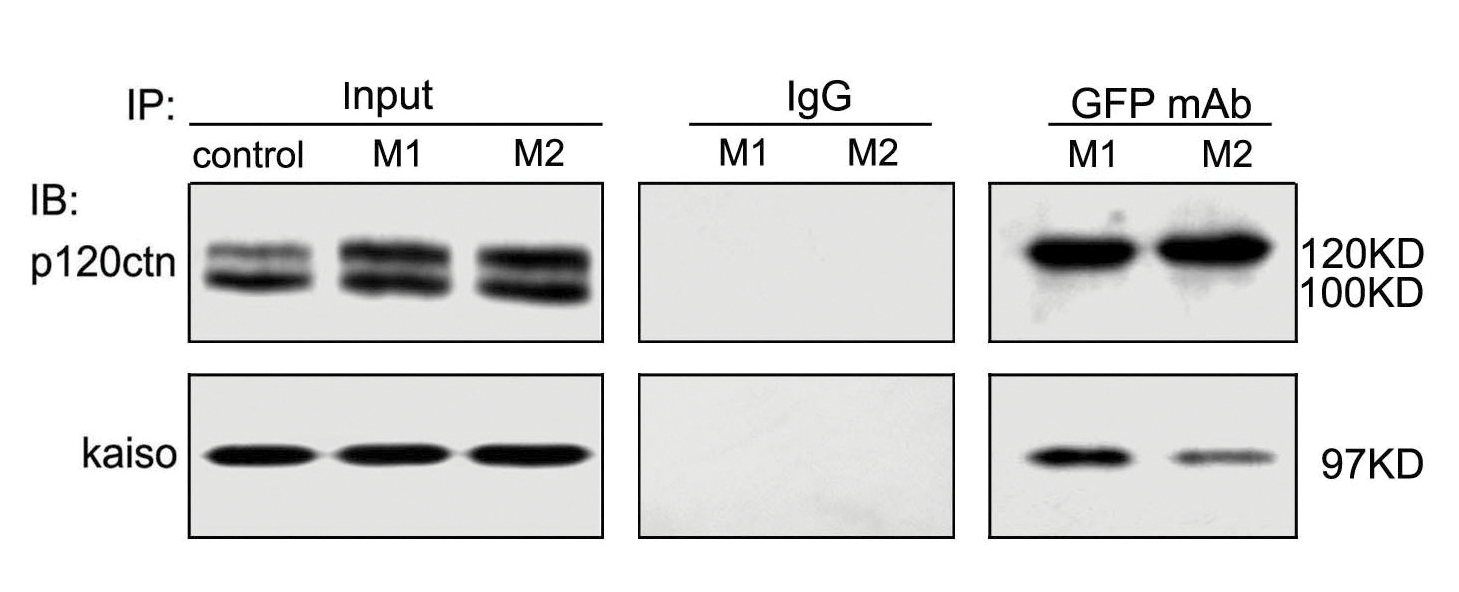

Supplement: Figure S9 — Co-immunoprecipitation was carried out following transfection of M1 or M2 with sufficient protein and equivalent GFP monoclonal antibody. The GFP monoclonal antibody could effectively precipitate exogenous deletion mutants of p120ctn-1 and Kaiso protein, and the binding affinity of M1 seems to be significantly stronger than M2, implying the coiled coil domain, located in the N-1-55 amino acid residues of p120ctn isoform 1, might prevent p120ctn isoform 1 from binding to Kaiso. (TIF) [file pone.0030303.s009.tif]

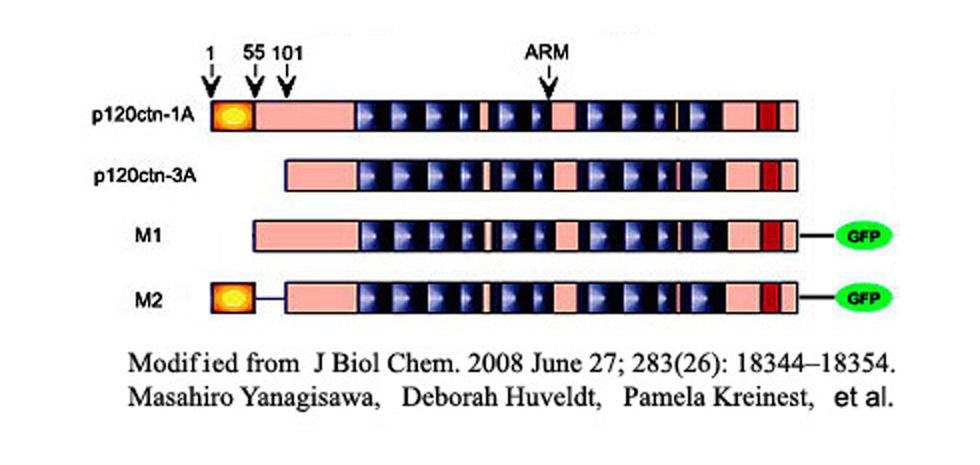

Supplement: Figure S10 — Two deletion mutants of p120ctn-1 M1 and M2 lack N-1-55 amino acid residues and N-56-101 amino acid residues respectively. (TIF) [file pone.0030303.s010.tif]
